# Sex-Determination System in the Diploid Yeast *Zygosaccharomyces sapae*

**DOI:** 10.1534/g3.114.010405

**Published:** 2014-06-01

**Authors:** Lisa Solieri, Tikam Chand Dakal, Paolo Giudici, Stefano Cassanelli

**Affiliations:** Department of Life Sciences, University of Modena and Reggio Emilia, 42122, Reggio Emilia, Italy

**Keywords:** homothallism, mating-type evolution, chromosomal rearrangement, HO endonuclease, genetics of sex, sex chromosome

## Abstract

Sexual reproduction and breeding systems are driving forces for genetic diversity. The mating-type (*MAT*) locus represents a mutation and chromosome rearrangement hotspot in yeasts. *Zygosaccharomyces rouxii* complex yeasts are naturally faced with hostile low water activity (a_w_) environments and are characterized by gene copy number variation, genome instability, and aneuploidy/allodiploidy. Here, we investigated sex-determination system in *Zygosaccharomyces sapae* diploid strain ABT301^T^, a member of the *Z. rouxii* complex. We cloned three divergent mating type-like (*MTL*) α-idiomorph sequences and designated them as *ZsMTL*α copies 1, 2, and 3. They encode homologs of *Z. rouxii* CBS 732^T^ MATα2 (amino acid sequence identities spanning from 67.0 to 99.5%) and MATα1 (identity range 81.5–99.5%). ABT301^T^ possesses two divergent *HO* genes encoding distinct endonucleases 100% and 92.3% identical to *Z. rouxii HO*. Cloning of *MAT***a**-idiomorph resulted in a single *ZsMTL***a** locus encoding two *Z. rouxii*-like proteins MAT**a**1 and MAT**a**2. To assign the cloned *ZsMTL*α and *ZsMTL****a*** idiomorphs as *MAT*, *HML*, and *HMR* cassettes, we analyzed their flanking regions. Three *ZsMTL*α loci exhibited the *DIC1-MAT-SLA2* gene order canonical for *MAT* expression loci. Furthermore, four putative *HML* cassettes were identified, two containing the *ZsMTL*α copy 1 and the remaining harboring *ZsMTL*α copies 2 and 3. Finally, the *ZsMTL***a** locus was 3′-flanked by *SLA2*, suggesting the status of *MAT* expression locus. In conclusion, *Z. sapae* ABT301^T^ displays an aααα genotype missing of the *HMR* silent cassette. Our results demonstrated that mating-type switching is a hypermutagenic process in *Z. rouxii* complex that generates genetic diversity *de novo*. This error-prone mechanism could be suitable to generate progenies more rapidly adaptable to hostile environments.

Sexual reproduction is ubiquitous in eukaryotic organisms, from yeasts to human (Hadany and Comeron 2008). Hemiascomycetes in particular have evolved homothallic and heterothallic repertoires of bipolar mating strategies orchestrated by a single *MAT* locus, encoding key transcription factors that govern sexual identity and compatibility (Fraser and Heitman 2003). In contrast, other yeasts, such as *Candida albicans*, have developed alternative cryptic sexual cycle governed by a same-sex mating. The variability in mating system and sex chromosome may drastically affect population genetic structure, pathogen evolution, and ecological processes of survival and adaptation (Fraser and Heitman 2003; Bubnick and Smulian 2007; Hsueh *et al.* 2008), offering an in-deep understanding of factors that shape sex evolution, one of the major challenges in biology (Billiard *et al.* 2012).

In the haplo-diplontic yeast *Saccharomyces cerevisiae*, the *MAT* locus is located in centromeric region of chromosome III (CEN-*MAT* linkage) in two versions (idiomorphs), either *MAT***a** or *MATα* genes, enabling yeast to specify three cell types: haploid a, haploid α, and diploid a/α. In heterothallic strains of *S. cerevisiae*, mating takes place between cells bearing complementary *MAT* idiomorphs. However, *S. cerevisiae* exists in nature mainly as homothallic diploid strains (Mortimer 2000; reviewed in Greig and Leu 2009), and sexually reproduces in clonal cell populations by meiosis followed by mother–daughter mating (also referred to as haplo-selfing) (Knop 2006). A cassette model for mating-type switching has been proposed and further experimentally verified to explain haplo-selfing in *S. cerevisiae* (Hicks *et al.* 1977; Herskowitz 1988; Herskowitz *et al.* 1992). Mating-type switching is a programmed DNA rearrangement process that occurs in haploid budded cells and converts *MAT***a** into *MATα*, or vice versa (Strathern *et al.* 1982; Haber 1998). During switching, DNA at the *MAT* locus is removed and replaced with DNA copied from the heterochromatic silent cassettes near the telomeres of the chromosome III, either *HML* or *HMR*. The gene conversion is mediated by a LAGLIDADG homing endonuclease (HO), which catalyzes a site-specific double-strand break (DSB) at the boundary between the Y sequences unique to the *MAT*α or *MAT***a** alleles and the shared flanking Z sequences (reviewed in Haber 2012).

Based on comparative genomic analyses, the HO-catalyzed homothallic switching in the family Saccharomycetaceae arose from an obligate heterothallic ancestor system via a two-step process: (i) the origin of the silent cassettes (after the divergence of family Saccharomycetaceae from other families such as Debaryomycetaceae and the *Candida albicans* clade); (ii) the recruitment of *HO* gene, after the occurrence of a whole-genome duplication (WGD) event that split off the Saccharomycetaceae into the pre-WGD and post-WGD species, respectively (Wong *et al.* 2002; Butler *et al.* 2004). Despite the conservation of *HML* and *MAT* in *cis*, and of the α genotype at *HML*, the family Saccharomycetaceae displays consistent variability in idiomorph content and chromosomal organization at the *MAT* locus (Tsong *et al.* 2003; Butler *et al.* 2004; Fabre *et al.* 2005; Gordon *et al.* 2011). Unlike *S. cerevisiae* and closest relatives, other yeasts have no constrained *HMR* linked to *MAT* and *HML* loci on sex chromosome (Fabre *et al.* 2005). Moreover, the *S. cerevisiae MAT* loci code for only three proteins (the homeodomain proteins **a**1 and α2 and the “α-domain” protein α1), whereas an additional gene (*MAT***a**2) coding for an HMG domain DNA-binding protein is present in the *MAT***a** idiomorph of several species (Butler *et al.* 2004). Almost all the pre-WGD species retain a stable chromosomal organization with a restricted set of ancestrally conserved genes flanking the *MAT* locus. On the contrary, in post-WGD species the *MAT* locus is subjected to a continual process of erosion, leading different genes incorporated into the Z and X regions, making the sex chromosome a hotpsot for deletion and transposition (Martin *et al.* 2010; Gordon *et al.* 2011).

The protoploid yeast *Zygosaccharomyces rouxii* is one of the few pre-WGD species that split off from post-WGD species after the gain of *HO* gene (Butler *et al.* 2004). *Z. rouxii* strains commonly inhabit low a_w_ environments and have been used for centuries as fermented food starters for the production of sugary and salty food, but they can also determine food spoilage, which accounts for huge economical loss to food industry (Solieri and Giudici 2008; Dakal *et al.* 2014). *Z. rouxii* traditionally has been considered as a predominantly haploid yeast with a bipolar mating system (Wickerham and Burton 1960). Because sporulation requires a diploid DNA content, the species with a haploid lifestyle, such as *Z. rouxii*, must first undergo mating between heterothallic a and α cells in response to osmostress. The resulting transient a/α diploid zygote usually enters in meiosis, producing from two to four haploid gametes. Syngamy of homothallic strains is also possible between genetically identical haploid cells by mating-type switching, followed by meiosis to restore the haploid status. Remarkably, alternative modes of reproduction have been observed but poorly investigated. For example, cell fusion could be not followed by nuclear fusion, resulting in a dikaryon that produces haploid buds (Mori 1973). In addition, zygote may lose the meiotic ability and begins clonal euploid/aneuploid lineages (Solieri *et al.* 2013a). Indeed, *Z. rouxii* in yeast culture collections have been demonstrated considerable variation in ploidy and karyotype (James *et al.* 2005; Gordon and Wolfe 2008; Solieri *et al.* 2008, 2013a,b), that corresponds to phenotypic variability in survival under stress cues (Solieri *et al.* 2014).

Based on these evidences, at least three groups have been delineated and globally referred to as *Z. rouxii* complex: the group of haploid *Z. rouxii*, including the strain CBS 732^T^, an allopolyploid group composed of strain ATCC 42981 and aneuploid relatives, and the novel diploid species *Zygosaccharomyces sapae*, which display mainly a clonal reproduction and rarely goes through meiosis resulting in ascospores (Gordon and Wolfe 2008; Solieri *et al.* 2013a,b). The coexistence in the same phylogenetic group of very closely related species of sexual and putative asexual taxa with similar ecological and physiologic properties raises several questions: (i) is *Z. sapae* truly asexual, having thus no traces of *MAT* genes in their genomes? (ii) Alternatively, has asexual species formed recently and, therefore, it still exhibits unfunctional sex related genes? (iii) Is mating-type imbalance possibly responsible for asexual lineages? Recently, the analysis of the *MAT* structure in haploid *Z. rouxii* strains revealed a remarkable rearrangement of sex chromosome by ectopic recombination, leading to strains with unusual genetic make-up ααα and αααα (Watanabe *et al.* 2013). These evidences support that sex chromosome is prone to nonhomologous recombination in *Z. rouxii* species complex. However, no evidences about the *MAT* loci organization have been reported in diploid lineages. In this study, we surveyed the presence and integrity of *MAT* and *HO* genes in *Z. sapae* diploid type strain ABT301^T^.

## Materials and Methods

### Strains and mating tests

The *Z. sapae* ABT301^T^ strain was retrieved from high sugary traditional balsamic vinegar (Solieri *et al.* 2006, 2013b) and deposited to the Yeast Collection of the Centraalbureau voor Schimmelcultures (CBS; Utrecht, The Netherlands) and to the Mycothéque de l’Université Catholique de Louvain (MUCL; Louvain-la-Neuve, Belgium) under the codes CBS 12607^T^ and MUCL 54092^T^, respectively. *Zygosaccharomyces rouxii* strains CBS 732^T^, CBS 4837 (mating-type a) and CBS 4838 (mating-type α) were achieved from CBS collection. Strains were cultured and maintained in the yeast extract-peptone-glucose medium (1.0% yeast extract, 1.0% peptone, and 2.0% glucose, w/v). To study sexual compatibility, 2- to 4-d-old cultures of ABT301^T^ were incubated alone or in mixture to *Z. rouxii* CBS 4837 or CBS 4838 both on malt extract agar (MEA; Difco) and MEA supplemented with 6% (w/v) NaCl (6%NaCl-MEA), at 27° for 2−3 wk and examined microscopically using phase-contrast optics for production of conjugated asci.

### Standard DNA manipulation

Genomic DNA (gDNA) was extracted from early stationary cultures via the phenol/chloroform method (Hoffman and Winston 1987). The restriction enzymes were purchased from Fermentas (Burlington, ON, Canada); rTAQ DNA polymerase and high-fidelity Phusion DNA polymerase from Takara (Takara Bio Inc., Shiga, Japan) and ThermoFisher (ThermoFisher Scientific, Waltham, MA), respectively; and the DNA ligation kit from Promega (Madison, WI). Plasmid preparations, polymerase chain reactions (PCRs), and other standard molecular biology techniques were performed as described elsewhere (Sambrook *et al.* 1989) or as instructed by suppliers. In particular, standard PCR mixtures (25–50 μL) contained 10 mM Tris-HCl (pH 8.3), 50 mM KCl, 1.5 mM MgCl_2_, 200 μM of each deoxynucleotide triphosphate, 0.4 μM each primer, 0.02 U/μL of rTaq DNA polymerase, and 100−200 ng of template DNA. The thermal program consisted of one cycle of 5 min at 94° followed by 35−40 cycles of 94° for 45 sec, 58° for 1 min, and 72° for 2 min. For amplification of DNA fragments >2 kb, PCR mixtures (20 μL) contained 1X Phusion HF Buffer, 200 μM each deoxynucleotide triphosphate, 0.5 μM each primer, 0.02 U/μL Phusion DNA polymerase, and 100−200 ng of template DNA. The thermal program consisted of 1 cycle of 98° for 1 min, 25−35 cycles of 98° for 10 sec, 60−68° for 30 sec, 72° for 30 sec/kb, followed by 1 cycle of 72° for 10 min. All the PCRs were performed with BioRad T100 Thermalcycler (Bio-Rad Laboratories, Hercules, CA). Primer design was performed using the Primer3 software (Untergasser *et al.* 2012). Screening of cloning libraries containing PCR products from degenerate primers were performed by sequencing at least three plasmids. All the sequencing reactions were carried out by a custom sequencing service provider (BMR Genomics, Padova, Italy)

### Cloning of *MAT* loci

**Figure 1:**
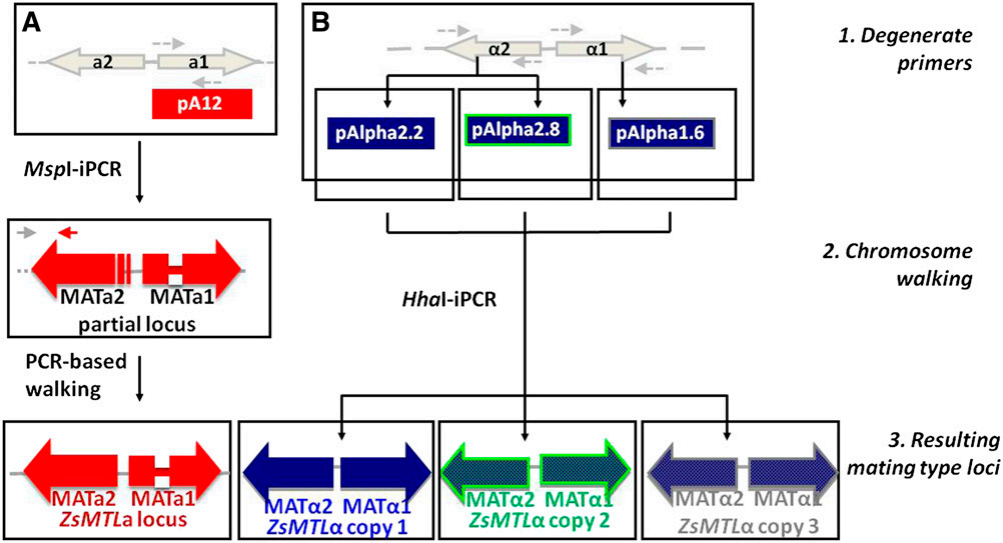
Outline of the cloning strategy and the resulting *Z. sapae* mating-type loci. Small horizontal arrows indicate degenerate primers (dotted) or gene-specific primers (solid). Dotted horizontal lines represent unknown genomic sequences. The discovered *ZsMTL*a and *ZsMTL*α loci are summarized in colored red and blue boxes, respectively. Divergent copies of *ZsMTL*α are surrounded by blue (copy 1), green (copy 2), and gray (copy 3), respectively. Abbreviation: iPCR, inverse PCR.

Schematic strategy of *MAT* idiomorphs cloning is outlined in Figure 1. To summarize, degenerate primers were designed based on a set of amino acid sequences that represent highly conserved regions of homologous proteins MAT**a**1, MATα1, and MATα2 from the species *S. cerevisiae* and *Z. rouxii* (Supporting Information, Table S1). These degenerate primer pairs were used to amplify via PCR similar conserved regions in *Z. sapae* gDNA. Individual gel bands from amplified *MAT*α1 and *MAT*α2 PCR products showing predicted sizes of 495 and 578 bp, respectively, were gel-extracted by using the Qiaquik column method (Qiagen) and cloned into pGEM-T Easy vector (Promega). Inserts from recombinant plasmids pAlpha2.2, pAlpha2.8, and pAlpha1.6 were submitted to sequencing in both directions with vector primers T7 and SP6. Similarly, *MAT***a**1-targeting degenerated primers were used to amplify via PCR a fragment of expected size of 153 bp, which was gel extracted and cloned as reported previously, resulting in a plasmid pA12 submitted to sequencing, as described above.

The *MAT* sequences were further extended by inverse PCR and PCR walking using plasmid partial sequences pAlpha2.2, pAlpha2.8, pAlpha1.6, and pA12 as starting points. To summarize, to extend *MAT*α and *MAT***a** sequences, gDNA (200 ng) was digested with *Hha*I and *Msp*I, respectively, and the resulting DNA digests were ligated with T4 DNA ligase (Promega). The digestion/ligation products were 10-fold diluted, 1 μL was used for 25 μL of PCR using rTaq polymerase (Takara), and the primers listed in Table S2. To complete *MAT***a**2 sequence, the primer 301_MATA2F1, spanning the 5′UTR region of *MAT***a**2 open reading frames (ORFs) in *Z. rouxii* ZYRO0C18326g locus was used together with an internal *MAT***a**2-specific primer (301_MATA2R1) in PCR amplification (Table S2).

### Cassette system determination

To verify whether the gene organization around *Z. sapae MTL* loci resembles those described in other yeast species (Butler *et al.* 2004; Watanabe *et al.* 2013), PCR amplification of gDNA was carried out by using primer sets spanning putative *MTL*-flanking genes (Table S3). To summarize, the first round of long-range PCR was done with high-fidelity DNA polymerase (Phusion, Thermofisher) and the external primers 1, 2, 3, A, B, B′, C (Watanabe *et al.* 2013), and DownMATa1R1 (this study) in 20 µL of reaction volume, following the manufacturer’s instructions. Subsequently, a seminested PCR amplification was done using a 1:20 dilution of the previous PCR and internal *MTL* locus-specific primers. In case of negative results in first round of PCRs, we tested alternative combinations of *MTL*-flanking genes by direct PCR amplifications from gDNA with the following primers sets: 1, 2, 3/reverse nested *MTL*-specific primer (for 5′ end flanking genes) and forward nested *MTL*-specific primers/A, B, B′, C, and DownMATa1R1 (for 3′ end flanking genes). Amplified products were purified using the DNA Clean & Concentrator-5 kit (Zymo Research, Irvine, CA) and sequenced with the same primers used in PCRs.

### Cloning of *HO* genes

Schematic outline of cloning strategy was reported in Figure S1. To identify highly conserved amino acid sequences, homology comparison among the HO proteins from the species *S. cerevisiae* (AAA34683; NP_010054) and *Z. rouxii* (ZYRO0C10428p), as well as the *S. cerevisiae* VMA intein (AAL18609) was performed by ClustalW2 alignment (Larkin *et al.* 2007). Relied on the resulting conserved motifs, two degenerate primer pairs, ZrHOF2/ZrHO_R2 and ZrHOF3/ZrHO_R3, were designed and used to amplify the N- and C-terminal coding regions of the putative *Z. sapae HO* gene, respectively (Table S1). PCR fragments of expected length were gel extracted and cloned as previously reported. The plasmids pHO2.3 and pHO2.8 bearing two inserts coding for putative HO N-terminal portions and pHO3.5 containing an insert covering the HO C-terminal portion were identified by sequencing in both directions. Genomic portions cloned in pHO2.3 and pHO2.8 were joined to the insert cloned in pHO3.5 by PCR amplifications with primers pairs 301_5′HOF1/301_5′HOR1 and 301_5′HOF3/301_5′HOR1, respectively (Table S2). The resulting two partial *HO* contigs were referred to as copy 1 and copy 2. Subsequently, the full-length ORF sequences of *HO* copies 1 and 2 were achieved by PCR-based walking. For upstream walking, a forward primer targeting the 5′ UTR of *Z. rouxii* CBS 732^T^ *HO* gene (ZYRO0C10428g) was combined with two *HO* copy-specific reverse primers (Table S2). The sequences flanking the 3′ ends of both copies were covered through a two-steps PCR walking strategy. In the first step, *HO* copy-specific forward primers were combined with degenerate reverse primer targeting the HO conserved domain FYRDWSG. In the second one, forward *HO* copy-specific primers were exploited together with a downstream reverse primer, targeting the 3′ UTR of *Z. rouxii HO* gene (Table S2).

### gDNA- and PFGE-based Southern blot assays

Southern blot assays were performed according to standard procedures described by Sambrook *et al.* (1989). gDNA (7 μg) was digested with the restriction enzymes listed in Table S4 following the manufacturer’s instructions and resolved on 0.8% (w/v) agarose gel in 0.5X TBE buffer. Chromosomal DNA preparation in plug, gel preparation, and pulsed-field gel electrophoresis (PFGE) were performed as previously reported (Solieri *et al.* 2008). Digested gDNA and chromosomal DNAs separated by PFGE were transferred onto a Hybond-N+ membrane (GE Healthcare, Buckinghamshire, UK) by upward capillary transfer. In both experiments, probe synthesis was performed using a PCR DIG probe synthesis kit (Roche Applied Science, Basel, Switzerland) and detection was carried out by chemiluminescence, using an antidigoxigenin antibody and CDP-star (Roche Applied Science) according to the manufacturer’s instructions. Primers engaged in probe synthesis and restriction enzymes for Southern blot assays were listed in Table S4.

### Sequence analysis, phylogenetic construction, and protein domain identification

Database searches were run with the BLAST server at the National Center for Biotechnology Information (http://www.ncbi.nlm.nih.gov/BLAST). Multiple sequence alignments were performed with the ClustalW program at the European Molecular Biology Laboratory (http://www.ebi.ac.uk/clustalw) and manually refined. Phylogenetic trees were constructed by the neighbor-joining (NJ) method using MEGA version 5.0 from ClustalW alignment (Tamura *et al.* 2011). Bootstrap support was estimated using 1000 pseudoreplicates for distance analysis. Statistics relating to the identification of Pfam domains of predicted proteins were obtained from PFAM protein family database, version 27.0 (Punta *et al.* 2012). Structure predictions were obtained with Jpred3 (Cole *et al.* 2008). Sequence data from this article have been submitted with the EMBL/GenBank Data libraries under accession numbers HG931712−HG931721.

## Results

### Mating test

We first assessed the mating behavior of ABT301^T^ strain in pure and mixed cultures with the *Z. rouxii* mating partners CBS 4837 (mating-type a) and CBS 4838 (mating-type α), respectively. Our previous observations show that ABT301^T^ rarely formed asci in pure culture on MEA medium, which involved mother and daughter cells that remained attached to each other (Solieri *et al.* 2013b). No conjugated asci were observed on 6%NaCl-MEA after 14 d of incubation. Furthermore, strain ABT301^T^ showed no mating reaction with *Z. rouxii* CBS 4837 or CBS 4838 tester strains, even after 3 wk of incubation both on MEA and 6%NaCl-MEA media (data not shown), suggesting the homothallic state for ABT301^T^ or that ABT301^T^ did not respond to *Z. rouxii* pheromone signaling or that its pheromone expression might be repressed or defective.

### Isolation and characterization of *Z. sapae MTL*α loci

**Figure 2:**
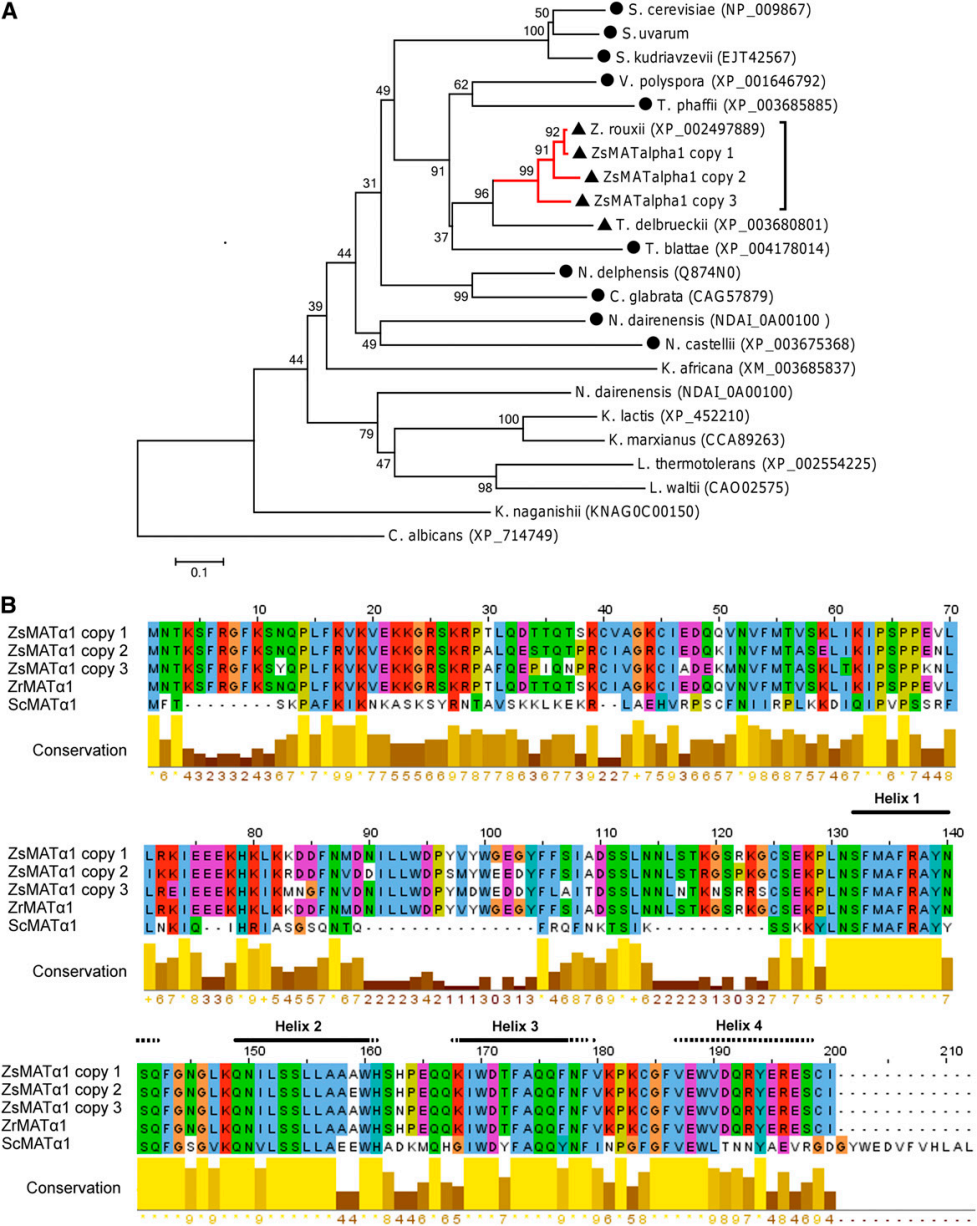
Phylogenetic analysis and sequences comparison of MATα1 proteins. (A) A neighbor joining (NJ) tree shows the phylogenetic relationships between *Z. sapae* and other hemiascomycetes inferred from MATα1 proteins. Number on branches indicates bootstrap support (1000 pseudoreplicates) from NJ. The red branch indicates ZsMATα1 sequences, the dark dot indicates post-WGD species, and the dark triangle indicates pre-WGD species with *HO* gene. (B) Amino acid alignment of putative MATα1 copies isolated from *Z. sapae* (ZsMATα1 copy 1, 2, and 3; GenBank: CDM87333, CDM87336, and CDM87339) and the orthologs from *Z. rouxii* (ZrMATα1; GenBank: XP_002497889) and *S. cerevisiae* (ScMATα1; GenBank: NP_009867). The helices that characterized the conserved MATα-HMG domain for mating-type proteins MATα1 (Martin *et al.* 2010) are shown: solid horizontal bars indicate common secondary structures between *Zygosaccharomyces* and *Saccharomyces* species, and dotted horizontal bars indicate *Saccharomyces*-specific secondary structures. The amino acid identities were colored according the ClustalX color scheme, and the conservation index at each alignment position were provided by Jalview (Waterhouse *et al.* 2009).

To determine how the mating-type information is retained in *Z. sapae* genome, we cloned the *MATα* loci from ABT301^T^ strain. Two degenerate primer pairs built on highly conserved regions of MATα1 and MATα2 were employed for cloning *MAT*α1 and *MAT*α2 ORFs, respectively. One putative MATα1-coding and two MATα2-coding partial sequences were obtained (Figure 1). Chromosome walking by inverse PCR and PCR was used to further extent these sequences, resulting in three divergent *Z. sapae* mating type-like loci α, referred to as Zs*MTL*α locus copy 1, copy 2, and copy 3 (Figure 1). Based on a BLAST-type search, two ORFs, namely *ZsMAT*α1 and *ZsMAT*α2, were predicted in each *ZsMTL*α locus, encoding proteins of 200 and 225 amino acid homologous to *Z. rouxii* MATα1 and MATα2, respectively, and separated by an intervening 343-bp sequence (Figure 1). All three *ZsMTL*α loci displayed an identical organization, with the *ZsMAT*α1 and *ZsMAT*α2 genes orientated in opposite direction on complementary DNA strands, suggesting a configuration similar to those found in *S. cerevisiae* and other hemiascomycetes (Butler *et al.* 2004). To establish the genomic location of *ZsMTL*α loci, we combined Southern blot analysis and PFGE-karyotyping. As previously reported (Solieri *et al.* 2008), PFGE-Southern blotting failed to clearly resolve the highest molecular weight chromosomes spanning from 1.6 to 2.2 Mbp, and labeled as I, L and L′, respectively (Figure S2). Hybridization of PFGE-Southern blot with an α-idiomorph specific probe resulted in a double band spanning from chromosome I and L, suggesting that *ZsMTL*α loci reside on at least two similar high molecular weight chromosomes. The coding regions of *ZsMTL*α loci were compared each others and with known sequences from *Z. rouxii* and *S. cerevisiae* orthologs (Table 1). The *ZsMAT*α1 genes from *ZsMTL*α copies 2 and 3 diverged from the *ZsMAT*α1 gene harbored in *ZsMTL*α copy 1 for 68 and 82 nt substitutions, respectively. The deduced proteins ZsMATα1 copies 2 and 3 were 200-amino acid long and showed less percentage identities with the *Z. rouxii* counterpart compared to ZsMATα1 copy 1 (Table 1). The NJ-tree was constructed using a selection of MATα1 sequences from representative taxa of post and pre-WGD species. As expected, ZsMATα1 copies 2 and 3 did not group to *Z. rouxii* MATα1, but instead clustered separately (bootstrapping values of 91 and 99%, respectively), with copy 2 being closer to *Z. rouxii* MATα1 than copy 3 (Figure 2A).

**Table 1:** Identities based on nucleotide and amino acid sequences for *MAT*α1 and *MAT*α2 genes isolated from *Zygosaccharomyces sapae* ABT301^T^

*Z. sapae* α-Idiomorph Genes	Accession No.	Identity % (bp, aa)	
		*ZrMAT*α1	*ScMAT*α1
*ZsMAT*α1 copy 1	HG931712	99.8, 99.5	35.2, 32.0
*ZsMAT*α1 copy 2	HG931713	86.6, 87.5	35.8, 32.0
*ZsMAT*α1 copy 3	HG931714	83.9, 81.5	36.2, 30.9
		*ZrMAT*α2	*ScMAT*α2
*ZsMAT*α2 copy 1	HG931712	99.9, 99.5	42.4, 38.6
*ZsMAT*α2 copy 2	HG931713	77.6, 80.5	36.3, 39.1
*ZsMAT*α2 copy 3	HG931714	67.7, 67.0	37.1, 35.1

Zr, *Zygosaccharomyces rouxii*; Sc, *Saccharomyces cerevisiae*; Zs, *Zygosaccharomyces sapae*.

The alignment of ZsMATα1 copies with *Z. rouxii* and *S. cerevisiae* MATα1 proteins revealed the regions of highest similarity inside the MATα-HMG domain (Martin *et al.* 2010) and the acidic carboxyl terminal end (Figure 2B), whose integrity is required for DNA binding and vegetative incompatibility, respectively (Philley and Staben 1994). *Z. rouxii* MATα1 and all ZsMATα1 variants conserved the first three α helices predicted in *S. cerevisiae* (Martin *et al.* 2010), whereas they lacked the fourth alpha helix predicted at the C-terminus of S. cerevisiae MATalpha1. Searching in PFam-A database, we found that MATα-HMG domain from ZsMATα1 copy 2 adhered a little better to the consensus profile (PF04769; *E*-values 6.6e-09) than the homologous regions in copy 1 and 3 (*E*-value 1.8e-08 and 1.7e-08, respectively). For example, MATα-HMG domain in ZsMATα1 copy 2 had an E159 residue as in *S. cerevisiae* when in the same position this amino acid was replaced by alanine in ZsMATα1 copy 1 and 3, as well as in *Z. rouxii* MATα1. Although the consensus profile does not consider this substitution as conservative, it is still detectable in MATα-HMG domain of related species such as *Torulaspora delbrueckii*, *Vanderwaltozyma polyspora*, and *Candida glabrata*. In addition, the MATα-HMG domain of ZsMATα1 copy 3 displayed a H163N substitution compared with ZsMATα1 copies 1 and 2 and *Z. rouxii* MATα1. However, this position is poorly conserved in the consensus profile for MATα-HMG domain even inside Saccharomycetes. The amino acid substitutions among ZsMATα1 copies occurred mainly at their amino terminal ends, with the most divergent copy 3 displaying 17 unique residues, as well as 12 and 7 common substitutions with copy 2 and copy 1, respectively.

**Figure 3:**
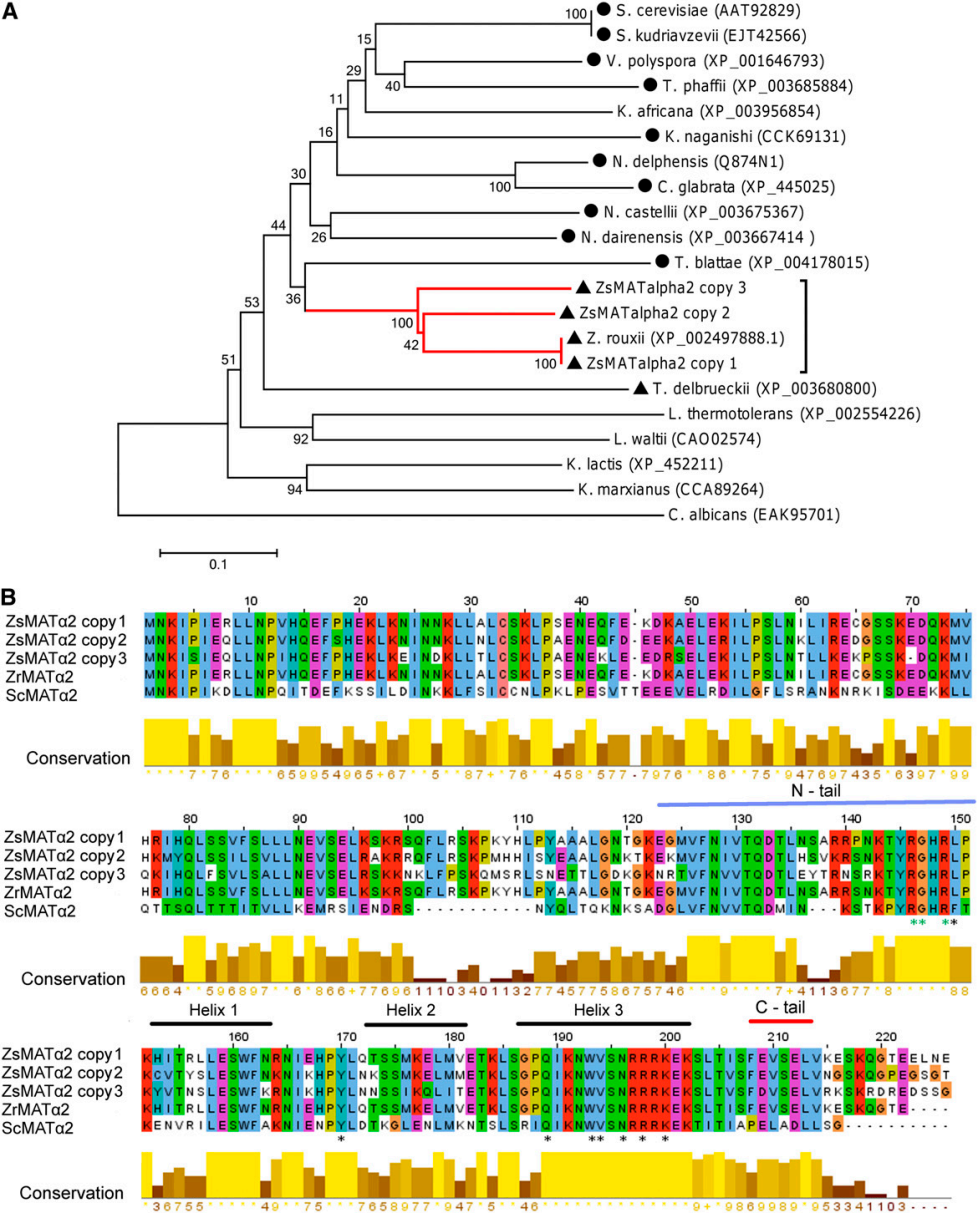
Phylogenetic analysis and sequences comparison of MATα2 proteins. (A) Neighbor-joining (NJ) phylogeny as inferred from MATα2 sequences depicting evolutionary relationships between *Z. sapae* and other hemiascomycetes. The number on branches indicates bootstrap support (1000 pseudoreplicates) from NJ. The red branch indicates ZsMATα2 sequences, the dark dot indicates post-WGD species, and the dark triangle indicates pre-WGD species with *HO* gene. (B) Alignment of deduced amino acid sequences from putative MATα2 genes cloned in *Z. sapae* (ZsMATα2 copy 1, 2, and 3; GenBank: CDM87332, CDM87335, and CDM87338) and orthologous MATα2 annotated in *Z. rouxii* (ZrMATα2; GenBank: XP_0024978881) and *S. cerevisiae* genomes (ScMATα2; GenBank: NP_009866). The *S. cerevisiae* DNA binding homeodomain of MATα2 (Pfam PF00046) consisting in three three-helix globular domains that contact major groove bases and the DNA backbone are indicated by horizontal black bars (Wolberger *et al.* 1991). Evolutionary conserved key residues involved in DNA binding are highlighted with black asterisks. Green asterisks denote amino acids that take additional interactions with the DNA minor groove in *S. cerevisiae* MATα2, present in the unstructured tail at the N-terminal side of homeodomain (light blue bar). The unstructured carboxy-terminal tail of *S. cerevisiae* MATα2 required for formation of a stable a1/α2-operator complex is also shown (red bar).

The MATα2 coding sequences from *ZsMTL*α loci copies 1, 2, and 3 showed 99.9, 77.6, and 67.7% of identities with *Z. rouxii MAT*α2 ortholog, respectively (Table 1). Phylogeny inferred from the MATα2 amino acid sequences of post and pre-WGD species showed a tree topology congruent with the species relationships established by using the MATα1 sequences. ABT301^T^ genome harbors three MATα2 variants, one (copy 1) clustered with *Z. rouxii* MATα2, whereas the others (copies 2 and 3) were related but phylogenetically distinct because of a high level of amino acid divergence (Figure 3A). All three copies contained a conserved HD1 class homeodomain (HD; Pfam PF00046; *E*-values 4.1e-7, 2.0e-7, and 9.5e-8, for ZsMATα2 copy 1, copy 2, and copy 3, respectively), consisting in a three-helix globular domain which contacts both major groove bases and the DNA backbone (Wolberger *et al.* 1991; Kues and Casselton 1992) (Figure 3B). Seven residues in helix 3 that contact the backbone with their side chains in *S. cerevisiae* MATα2 homeodomains also were conserved in *Z. rouxii*, *Z. sapae* along with the tyrosine residue (Y10 in *Z. rouxii* MATα2 just upstream at N-terminal of helix 1; Figure 3B). A further key tyrosine residue with the same structural role in *S. cererevisiae* MATα2 was indeed replaced by lysine in *Z. rouxii* (Y150L). The three residues of *S. cerevisiae* MATα2, which form additional interactions with the DNA minor groove, were conserved both in *Z. sapae* and *Z. rouxii* (R146, G147 and R149) (Ke *et al.* 2002). However, portions of the protein outside the homeodomain which mediate interactions with accessory proteins had a different degree of conservation. The unstructured carboxy-terminal tail of α2 is required for formation of a stable al/α2-operator complex in *S. cerevisiae* and, thus, for the heterodimer-mediated repression of transcription. This domain is fully conserved in *Z. sapae* and *Z. rouxii* MATα2 and largely resembled that found in *S. cerevisiae* (Mak and Johnson 1993). The intervening flexible hinge that connects the amino-terminal domain and the homeodomain of *S. cerevisiae* MATα2 mediates the interaction of MATα2α2 homodimer with two subunits of MCM1 and hence its operator binding capacity (Vershon and Johnson 1993). This sequence is more divergent in ZsMATα2 copy 3 compared with ZsMATα2 copies 1 and 2, and between MATα2 proteins in *Z. rouxii* and *S. cerevisiae*. The ability of MATα2 to form both homodimers (α2/α2) and heterodimers (α2/a) mainly relies on the integrity of the N-terminal portion (Ho *et al.* 1994, 2002). N-terminal homology between MATα2 in *Zygosaccharomyces* species and *S. cerevisiae* is less than that found for the homodomains, probably revealing a species-specific coevolution of the dimerization binding motifs. ZsMATα2 copy 3 was the most divergent from copies 1 and 2 (Figure 3A), owing to unique amino acid replacements even if, in a few positions, the residue was different in all three copies (Figure 3B), suggesting that these amino acid substitutions were less affected by functional constrains.

### Isolation and characterization of the *Z. sapae MTL*a locus

**Figure 4:**
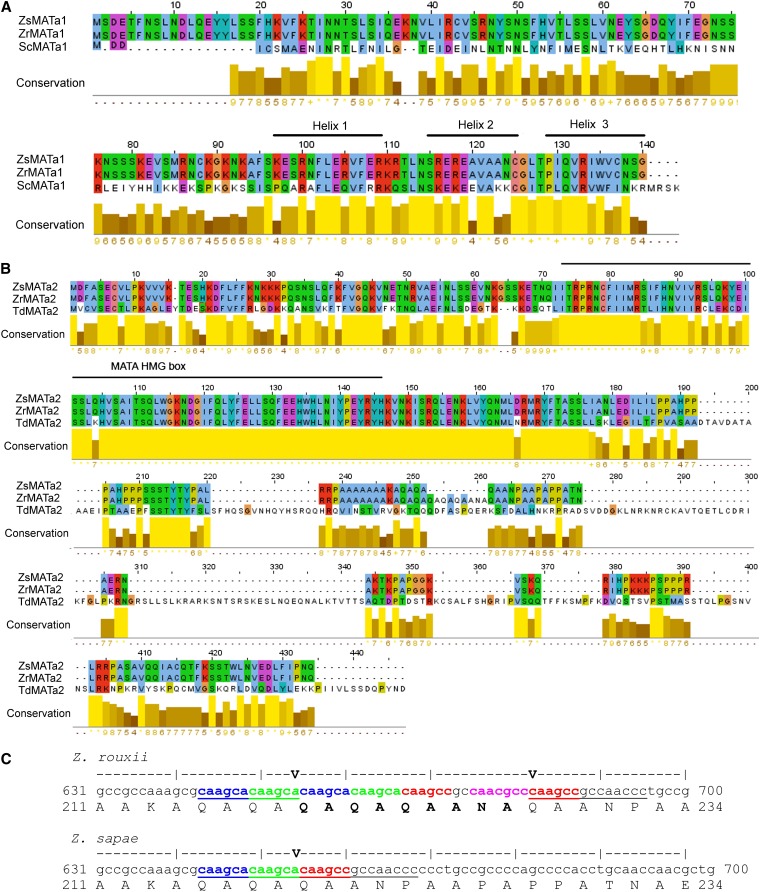
Amino acid sequence alignments of MATa1 and MATa2. (A) Alignment of MATa1 from *Z. sapae* (ZsMATa1, GenBank CDM87353), *Z. rouxii* (ZrMATa1; GenBank: XP_002496431), and *S. cerevisiae* (ScMATa1; GenBank: NP_010021). The three alpha helices that characterize the homeodomain (HD2 type) are highlighted (horizontal black bar). (B) Alignment of MATa2 from *Z. sapae* (ZsMATa2; GenBank CDM87352), *Z. rouxii* (ZrMATa2; GenBank: XP_002496430), and *Torulaspora delbrueckii* (TdMATa2; GenBank: XP_003682598). The MATA HMG domain, which binds the minor groove of DNA, is noted (horizontal black bar). In both alignments, the amino acid identities were colored according the ClustalX color scheme and the conservation index at each alignment position were provided by Jalview (Waterhouse *et al.* 2009). (C) Partial nucleotide sequence alignment shows indel junction boundaries (V) in *Z. rouxii* and *Z. sapae* MATa2. Imperfect tandem repeat units are highlighted in different colors.

A strategy similar to that used for cloning *ZsMTL*α loci, was carried out to isolate the *MAT***a**-like locus from *Z. sapae* genome (Figure 1). We obtained one single 1641-bp Zs*MTL***a** locus, which included two ORFs encoding putative MAT**a**1 and MAT**a**2 proteins, respectively, separated by a 279-bp intergenic sequence. The 474 bp MAT**a**1-coding ORF, namely Zs*MAT***a**1, displayed a putative 51-bp intron and resulted in a deduced ZsMAT**a**1 140-aa sequence 100% identical to *Z. rouxii* MAT**a**1 (Figure 4A). With respect to genomic location, PFGE-Southern blotting showed that *ZsMTL***a** locus resides on the single high molecular weight chromosome L poorly resolved from chromosome L′ (Figure S2B). The MAT**a**1 harbored a conserved HD2 class homeodomain (Pfam *E*-value, 8.1-e10, PF00046), consisting of an unstructured N-terminal arm and three helices linked by two loops (Figure 4A) (Kues and Casselton 1992; Anderson *et al.* 2000).

The MAT**a**2-coding ORF, namely Zs*MAT***a**2, was shorter in length that the *Z. rouxii* ortholog (ZYRO0C18326g) due to a 26-bp deletion. Thus, the deduced ZsMAT**a**2 amino acid sequence is 9 amino acids shorter than *Z. rouxii* MAT**a**2 and lacks the domain _219_(QAQAQAANA)_227_ (Figure 4, B and C). MAT**a**2 was provided with single MATA_HMG-box, class I member of the HMG-box superfamily of DNA-binding proteins (NCBI’s Conserved Domain Database code: cd01389; residues 72-145; *E*-value 4.31e-06; Figure 4B), coding by a sequence spanning across Y**a** and X regions. Beyond this putative functional domain, there were a very few spotted similarities with MAT**a**2 annotated in close related species. The inferred joint point responsible for peptide removal from MAT**a**2 in *Z. sapae* laid on X region and went through an imperfect tandem sequence (CAAGCA/C)_3_ at the nucleotide position 653 (Figure 4C).

### System cassette analysis

**Figure 5:**
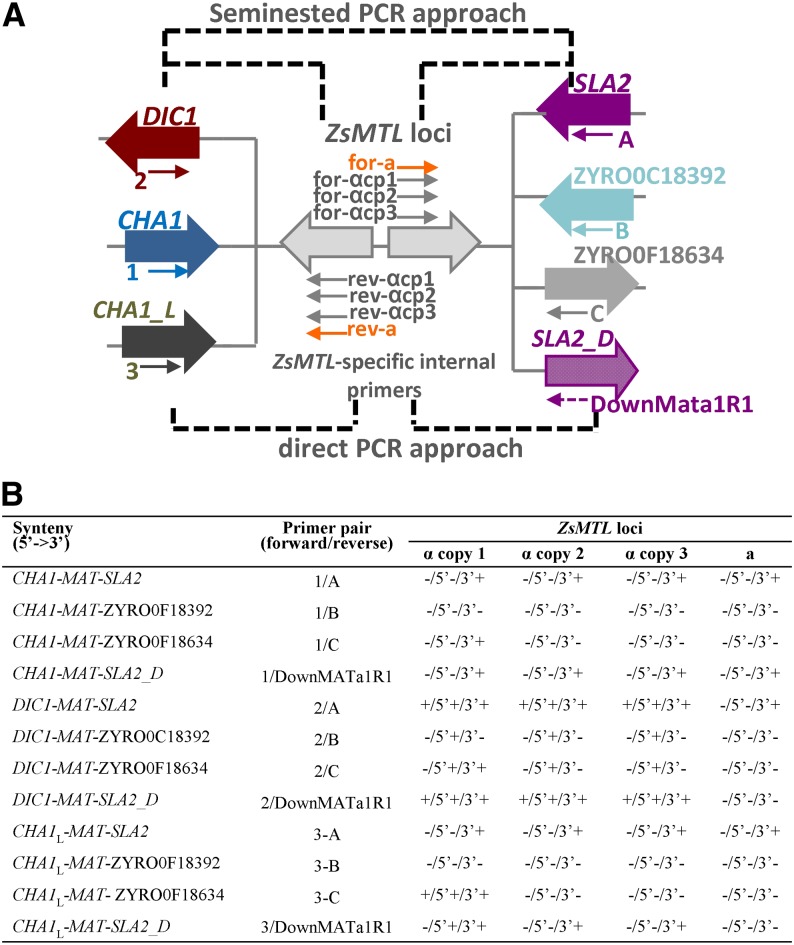
Variability of *MTL*-flanking regions in *Z. sapae* ABT301^T^. (A) Polymerase chain reaction (PCR)-based strategies used for determining chromosomal three cassette system in *Z. sapae*. Forward and reverse *ZsMTL*-specific internal primers were used to screen PCR products obtained by using all possible combinations of primers spanning putative *MTL*-flanking genes (seminested PCR approach); in case of negative results, 5′ and 3′ PCR walking was done by using all possible combinations of *ZsMTL*-specific internal primers and *MTL*-flanking genes primers (direct PCR approach). Small arrows indicate gene-specific primers (solid lines) and degenerate primer (dotted lines). (B) PCR amplification results of the Zs*MTL* loci from ABT301^T^. Left symbols indicate presence or absence of PCR products after seminested PCR; middle symbols indicate presence or absence of direct PCR products at 5′ end; right symbols indicate presence or absence of direct PCR products at 3′ end. *SLA_D*, divergent *SLA2* gene partial sequence determined with the degenerate primer DownMATa1R; *CHA1*_*L*, *CHA1* (ZYRO0F18524) located near to the silent *HML* cassette in CBS 732^T^ genome; cp, copy. All primer sequences are in Table S3.

In *S. cerevisiae*, the functional *MAT*α locus is flanked by *BUD5* at the 5′ end of *MAT*α2 and by *TAF2* at the 3′ end of *MAT*α1, whereas the silent *HMR* and *HML* loci are flanked by YCRWDDta12/YCR097W-a and YCL068C/HCl065W, respectively. In *Z. rouxii*, several chromosomal arrangements have been revealed in different strains or in different collection cultures of the same strain (Watanabe *et al.* 2013), suggesting that the *MAT* locus is an ectopic recombination hotspot. The analysis of 3′ end flanking genes showed that *SLA2* gene is frequently linked both to *MAT* and *HML* cassettes in all the chromosomal rearrangements described in *Z. rouxii* (Watanabe *et al.* 2013) and in other hemiascomycetes (Gordon *et al.* 2011). To assign chromosomal positions and establish neighboring genes of *ZsMTL* idiomorphs, PCR amplifications across the whole cloned cassettes were performed by employing primer sets designed on genes flanking all *MAT*, *HML*, and *HMR* cassettes observed in other *Z. rouxii* strains (Figure 5A). To capture possible divergent sequences of 3′ end flanking regions of cloned *ZsMTL* idiomorphs, we designed a further degenerate primer DownMATa1R, spanning the motif FEFYADC of *Z. rouxii SLA2* gene (ZYRO0F15862g) (*SLA2_D*). Positive PCR products were obtained with the primer pairs 2/A, 3/C, 2/DownMATa1R and further screened via seminested approach using primers specific for *ZsMTL***a**, *ZsMTL*α copies 1, 2, and 3, respectively (Figure 5A).

No PCR products were gained with the primer 1 on *CHA1* (ZYRO0F15774g) gene sequence at *MAT*. To exclude alternative combinations of flanking genes other than those described by Watanabe *et al.* (2013), direct PCR was performed combining *ZsMTL*-copy specific primers and primers laying on other potential flanking genes (Figure 5A). The results of both approaches are reported in Figure 5B. A total of seven mating-type α cassettes were detected. Four were arranged in the following gene order: *CHA1_L* (ZYRO0F18524g)-*ZsMTL*α copy 1-*SLA2* (ZYRO0F18364g); *DIC1-ZsMTL*α copy 1-*SLA2* (ZYRO0F15862g); *DIC1-ZsMTL*α copy 2-*SLA2* (ZYRO0F15862g); and *DIC1-ZsMTL*α copy 3-*SLA2* (ZYRO0F15862g). The arrangement *CHA1_L* (ZYRO0F18524g)-*ZsMTL*α copy 1-*SLA2* (ZYRO0F18364g) is consistent with the designation of this locus as silent *HML* cassette (*ZsHML* copy 1) (Watanabe *et al.* 2013). The arrangement *DIC1-MAT-SLA2* indicates that three α-idiomorph *ZsMTL* cassettes are orthologous to *MAT* expression loci in other pre-WGD species and thus they are labeled as *ZsMAT*α copies from 1 to 3. Furthermore, the 2/DownMATa1R PCR amplicons were positive to all three *ZsMTL*α copy-specific internal primers, resulting in three additional α-idiomorphs cassettes. These cassettes had a *Z. rouxii DIC1*-like upstream region and a downstream region (*SLA2_D*) divergent from those found at 3′ ends of *Z. rouxii MAT* and *HML* loci (*SLA2* gene and ZYRO0F18524g locus, respectively). Based on the syntenic pattern *DIC1-ZsMTL*α-*SLA2_D*, these *ZsMTL*α cassettes were referred to as *ZsHML*_*D* copies 1, 2, and 3. Southern blot hybridization on digested gDNA with a *ZsMAT*α1 probe confirmed that at least seven mating-type α cassettes are present in ABT301^T^ (Figure S3A). Finally, the downstream region of the *ZsMTL***a** locus resulted to be orthologous to the *Z. rouxii SLA2* gene (ZYRO0F15862g). However, the gene at its 5′ end was still unknown, since all the PCR amplifications failed. This result suggested that the *ZsMTL***a** could be a *MAT*a expression locus in ABT301^T^ with an upstream genomic region not conserved between *Z. rouxii* and *Z. sapae*. The presence of a single mating-type **a**-idiomorph locus was also confirmed by gDNA-based Southern blotting (Figure S3B).

### Analysis of Z and X regions

In *Saccharomyces* species, the *MAT*, *HMR*, and *HML* cassettes share two homologous regions flanking the Y sequences, termed X and Z, which are regarded among the most slowly evolving sequences in the yeast genome (Kellis *et al.* 2003). Because HO creates a DSB within *MAT* locus at the junction between Y and Z sequences (Haber 1998), single base substitutions at the region near the Y/Z border are sufficient to inhibit *HO*-cut *MAT* switching (Weiffenbach *et al.* 1983; Nickoloff *et al.* 1986). To infer the functional state of Z sequences, we determined the extent of the sequence homology in the 3′ flanking regions of the eight *Z. sapae* mating-type cassettes (three *ZsHML*_*D*, three *ZsMAT*α, one *ZsHML* copy 1, and one *ZsMAT***a**, respectively). As expected in species with HO endonuclease, Y–Z junction was conserved in *ZsMAT***a**1 and all *ZsMAT*α1 genes. The eight *Z. sapae* mating-type cassettes were always found with the HO site-consensus sequence CGCAGC at the first site of the Z regions. This sequence was also found in *C. glabrata* (Butler *et al.* 2004) and represents a variant of the canonical *S. cerevisiae* recognition sequence (CGCAAC) for the HO site-specific enzymatic cleavage of *MAT* during switching (Figure S4). Both HO site-specific sequences have been shown to be cleaved efficiently by the *S. cerevisiae* HO *in vivo* (Nickoloff *et al.* 1990). The high level of conservation at the Y/Z borders suggests that all the mating-type cassettes could be functional either as putative *MAT* or *HML*/*HMR* donor sequences. Otherwise, base substitutions were observed at the 3′ end of Z region. In particular, four mating-type cassettes flanked by *Z. rouxii*-like 5′ and 3′ regions, namely *ZsHML* copy 1, *ZsMATα* copies from 1 to 3, and *ZsMAT***a**, displayed the Z regions 100% identical to those found in haploid *Z. rouxii* CBS 732^T^, whereas the Zs*HML_D* copies 1, 2, and 3 differed for 8 SNPs from the canonical *Z. rouxii* Z sequences. Finally, X region analysis showed that *ZsMAT***a**2 extends into the X region, whereas the X/Yα junction is upstream the codon stop of *ZsMAT*α2 genes. Consistently to this organization, the X regions in six *Z. sapae* α and one **a**-idiomorphs loci differ from those found in *Z. rouxii* and *ZsHML* copy 1 for the same 26-bp indel previously described in *ZsMAT***a**2 gene (Figure S5).

### Cloning of *HO* genes

**Figure 6:**
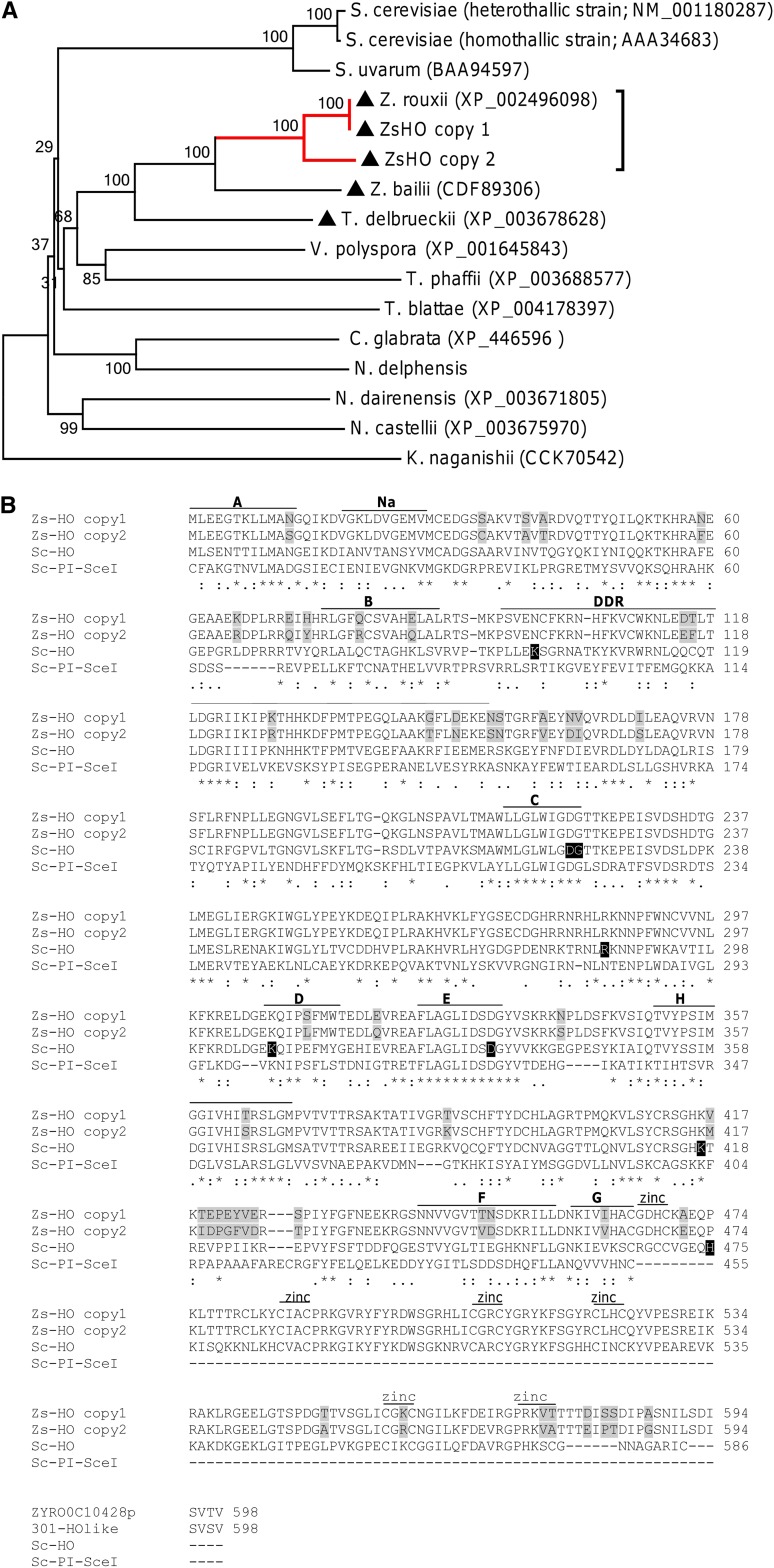
Phylogenetic analysis and amino acid sequences comparison of HO endonucleases. (A) Neighbor-joining (NJ) phylogeny as inferred from HO sequences depicting evolutionary relationships between *Z. sapae* and other hemiascomycetes. Numbers on branches indicate bootstrap support (1000 pseudoreplicates) from NJ. The Red branch indicates clusters, including ZrHO and ZsHO sequences, and the dark triangle indicates pre-WGD species. (B) Functional domains in PI-SceI and HO endonucleases. Primary amino acid alignment of *S. cerevisiae* PI-SceI (Sc-PI-SceI; GenBank: AA98762) and HO cloned in *S. cerevisiae* (Sc-HO; GenBank: CAA98806) and *Z. sapae* (ZsHO copy 1, GenBank: HG931720; ZsHO copy 2, GenBank: HG931721). Protein splicing domain with Hint motifs: A, Na, B, F, and G. Endonuclease domains C, D, E, and H. The DNA recognition region (DDR) and C-X2-C amino acid repeats (zinc) of putative zinc finger motifs at Sc-HO carboxyl-terminal are also shown (Bakhrat *et al.* 2004). In black shading are amino acid positions that are inferred to be critical for Sc-HO activity by analyzing naturally occurred or artificially induced HO mutants or by homology modeling with PI-SceI (Meiron *et al.* 1995; Ekino *et al.* 1999; Bakhrat *et al.* 2004; Ezov *et al.* 2010). In light gray, divergent positions between HO copies 1 and 2 in *Z. sapae* are shown. Amino acid identities are reported below the alignment following ClustalW rules: *, identity; :, conservative substitution; ., semiconservative substitution.

The occurrence of a *HO*-cleavable site in Z regions of all eight mating-type cassettes suggests that ABT301^T^ genome could harbor a *HO* endonuclease gene. To test this hypothesis, degenerate primer pairs were exploited to determine *Z. sapae* homologs of *Z. rouxii HO* gene (ZYRO0C10428g) (Figure S1). Two putative full-leght ORFs, namely *ZsHO* copy 1 and copy 2, were identified with 100 and 86.2% identities to *Z. rouxii HO* gene, respectively. The predicted *Z. sapae* HO proteins have 100 and 92.3% sequence identities to *Z. rouxii* HO protein. NJ-based phylogeny inferred from amino acid HO sequences showed that ZsHO copy 2 is clearly distinct from *ZsHO* copy 1 and *Z. rouxii HO* (Figure 6A). Southern blotting result on *Ban*II-digested gDNA with a probe able to recognize both *ZsHO*s was congruent with the occurrence of two gene copies in ABT301^T^ genome (Figure S3C). To determine the chromosome location of *HO* copies, we performed a PFGE-Southern blotting with the same probe. The results showed that the chromosomal position of *ZsHO*s differed from that of *Z. rouxii HO*. In *Z. rouxii* CBS 732^T^ the single *HO* gene is located on the low molecular weight chromosome C (Souciet *et al.* 2009), whereas in ABT301^T^ both *ZsHO* genes appear to be on the same high molecular weight chromosome I, which harbors the *ZsMTL*α loci (Figure S2C).

In *S. cerevisiae* homothallic strains, HO endonuclease is necessary to complete the sexual cycle by inducing the formation of cells with opposite mating-types within a clone. Because *Z. sapae* is unable to mate heterothallic sexual partner, its sexual reproduction may depend on the ability of some cells to switch mating-types and fuse with related cells. As shown in Figure 6B, the highest homology between both *HO* genes cloned in *Z. sapae* and the single *HO* genes found in *Z. rouxii* and *S. cerevisiae* corresponded to conserved motifs characteristic of intein-encoded LAGLIDADG endonucleases (Belfort and Roberts 1997; Stoddard 2005; Hafez and Hausner 2012). The two *Z. sapae* HO copies mostly differed in positions outside these functional domains (Figure 6B). With a few exceptions, *Z. sapae* HOs shared high identity in eight intein motifs lying at their C- and N- terminals, which form the relic of the protein-splicing domain in HO proteins. The intervening sequences around the LAGLIDADG motif in both ZsHOs were conserved and organized in four amino acid domains responsible for HO endonuclease activity. The C- terminal end of *S. cerevisiae* HO harbors three zinc finger domains thought enhancing the specificity of HO binding (Bakhrat *et al.* 2004). In ZsHOs these finger domains had the same organization in the primary sequence, even the last HX2C residue was absent. However, this motif can also be deleted from *S. cerevisiae* HO, without affecting the mating-type switching activity (Bakhrat *et al.* 2004).

Structural and mutagenesis studies of LAGLIDADG endonucleases, such as HO and PI-SceI in *S. cerevisiae*, revealed that the region downstream the B motif, the DDR region, although not well conserved in its primary sequence, probably contacts the phosphate DNA backbones of target site through charged lateral chains of key amino acid residues (He *et al.* 1998; Moure *et al.* 2002). This hypothesis was supported by the effect of K99A substitution in *S. cerevisiae* HO that abrogated the mating-type switching activity (Bakhrat *et al.* 2004). Indeed, there was high identity in the primary sequence of putative DDR regions in both *Z. sapae* HOs, whereas there was poor similarity with *S. cerevisiae* HO. Noteworthy, another positive charged amino acid (N97) was found in *Z. sapae* HOs instead of K99 residue found in *S. cerevisiae* HO. Similarly, both *Z. sapae* HOs conserved a few amino acid residues (*i.e.*, D222, G223, R286, K308, D333, K417), that, once replaced in *S. cerevisiae* HO, hampered the binding and/or endonuclease activities *in vivo* or *in vitro*, or are considered functionally relevant by homology modeling with PI-SceI (Meiron *et al.* 1995; Ekino *et al.* 1999; Bakhrat *et al.* 2004; Ezov *et al.* 2010). On the other hand, exceptions to this conservation were found. For example, residue H475 in *S. cerevisiae* HO, involved in DNA binding of endonuclease target sequence (Meiron *et al.* 1995; Ekino *et al.* 1999), was substituted by proline in both *Z. sapae* HOs.

## Discussion

Recently, nonconventional yeasts isolated from highly stress environments received enhancing attention both for biotechnological exploitation and genome evolution studies. Chronic osmotic stress triggers aneuploidy (Pfau and Amon 2012), increases the genome DNA content (Gerstein *et al.* 2006; Dhar *et al.* 2011), and favors chromosome instability (Aguilera and García-Muse 2013). The frequency of sex and the nature of breeding systems affect genome variation and adaptation to stress environments (Lee *et al.* 2010; Balloux *et al.* 2003). Although *Z. rouxii* and relatives are the most relevant osmo and halotolerant food yeasts, research into their mating systems is restricted to the haploid *Z. rouxii* strains (Butler *et al*. 2004; Gordon *et al.* 2011; Watanabe *et al.* 2013). Previous analysis demonstrated that *Z. sapae* diploid strains are genetically and phylogenetically distinct from *Z. rouxii* (Solieri *et al.* 2013a,b). Here, we examined mating-type system in *Z. sapae* strain ABT301^T^ and found that the pattern of *ZsMTL* loci is completely different from those described for haploid *Z. rouxii* strains. Based on genome project (Souciet *et al.* 2009), haploid strain CBS 732^T^ displayed the *MAT*α and *HML*α cassettes on chromosome F and the *HMR***a** cassette on chromosome C. The *MAT*α and *HML*α loci contain identical copies of *MAT*α1 and *MAT*α2 genes. Although this work was in progress, Watanabe *et al.* (2013) used a PCR-based method for tagging 5′ and 3′ *MAT*-flanking conserved regions in *Z. rouxii* haploid strains and in different cultures of the strain CBS 732T. This study revealed alternative interstrain arrangements in MAT loci and demonstrated a variable mating-type loci organization even in different cultures of the same strain.

Here, we exploited three experimental approaches, *i.e.*, *MAT* gene cloning, PCR *MAT* cassette placement, and PFGE-Southern blotting, to enroll the *MAT* loci cooccurring in ABT301^T^ genome and to inspect their genome configuration. First, we provided evidences that *Z. sapae* ABT301^T^ possesses four independent mating type-like loci, resulting in an unusual a,α,α,α genotype. In addition to one *ZsMTL***a** locus harboring *MAT***a**2 and *MAT***a**1 genes, we identified three *MTL*α loci, each containing pairs of *MAT*α1 and *MAT*α2 genes. Remarkably, in two of three *ZsMTL*α loci, *MAT*α1 and *MAT*α2 genes were slightly divergent from those described in the canonical *Z. rouxi MAT*α locus (ZYRO0F15840g and ZYRO0F15818g, respectively). A similar pattern of mating-type gene expansion has been recently found in *Hortaea werneckii*, a highly halotolerant and heterothallic black yeast, which possesses two divergent *MAT1-1-1* genes (Lenassi *et al.* 2013).

We hypothesize that the presence of three divergent *ZsMTL*α loci variants could be arisen from two alternative events. One route may consist in the amplification of a chromosomal segment containing the ancestral linked *MAT*α1 and *MAT*α2 genes, leading to paralogs that progressively accumulate mutations in the postduplication period. Potentially, this duplication could also involve the entire sex chromosome due to a chromosome missegregation during mitosis, which provides a diploid progeny with three chromosomes harboring progressively divergent *ZsMTL*α loci. In the second route, the acquisition of extra *ZsMTL* loci on homeologous sex chromosomes may take place after horizontal gene transfer (HGT) or interspecific introgression events. In fungi, interspecies *MAT* HGTs have been documented in clonal populations with increased adaptive phenotypes to new environments, but the underlying mechanisms are yet poorly understood (reviewed in Richards *et al.* 2011). Currently, there is no significant evidence to preferentially support one of the proposed alternatives about the generation of divergent *ZsMTL*α variants. Noteworthy, the amino acid sequence analysis reveals that substitutions among ZsMAT**a**1 or ZsMAT**a**2 copies are not randomly distributed. Accordingly, many residues crucial for transcriptional regulation activities of S. cerevisiae MATα1 and MATα2 are also conserved in the putative *Z. sapae* orthologs. These findings convey that divergent ZsMATα genes are under a selective driving force aimed to maintain functional integrity of the encoded transcription factors. The retention of three divergent and putatively functional *ZsMTL*α loci could be favored by the divergent transcription of *MAT*α1 and *MAT*α2 from the intervening promoter located on the intergenic region within each locus. Alternatively, the *ZsMTL*α extra loci could had been acquired from a close donor species through a very recent HGT event, limiting the sequence divergence among *ZsMTL*α copies.

The second goal was to establish whether *Z. sapae* has a *HO*/*MAT* cassette system like that in *S. cerevisiae* and *Z. rouxii*. Strain ABT301^T^ possesses two divergent *HO* genes, coding putatively functional endonucleases which share eight conserved intein motifs and the amino acid residues involved in DNA binding. Again these data hint that both the *ZsHO* genes are under the same selective pressure and that SNPs in *ZsHO* copies 1 and 2 are selectively neutral mutations, with negligible effects on gene function. However, *in vitro* switching tests are advisable to prove this hypothesis. Moreover, the high degree of divergence observed between *ZsHO* copies 1 and 2 suggests that these genes did not arise from a recent duplication event. Alternatively, they could result from a HGT event between two close yeast species, both bearing functional HO. All species that have *HO* genes have also silent cassettes (Butler *et al.* 2004). Although the post-WGD species contain highly variable organization of mating-type *MAT* locus and *HMR*/*HML* silent loci, the pre-WGD species retain the ancestral gene arrangement *DIC-MAT-SLA2* which distinguishes mating-type *MAT* locus from silent cassettes *HML* or *HMR* (Gordon *et al.* 2011).

To understand how the *ZsMTL* copies were organized in *MAT*, *HML*, and *HMR* cassettes, we explored the *ZsMTL* gene surroundings. One *MTL***a** and three *MTL*α variants have been anchored to flanking regions by PCR amplification using one primer specific to Yα (copy 1, 2, and 3, respectively) or Y**a** together with a primer annealing on common neighboring sequences found in *Z. rouxii* genome. Three *ZsMTL*α loci resulted duplicated in two syntenic patterns. One set, namely *ZsMAT*α copies 1, 2, and 3, exhibits the canonical synteny *DIC1-MAT-SLA2*. The other set includes three *ZsMTL*α loci with a gene layout *DIC1-ZsMAT*α-*SLA2_D*, regarded as *ZsHML*_*D*. The *ZsMTL*α copy 1 locus also fits to *CHA1-MAT-SLA2* gene organization (*ZsHML* copy 1). The *SLA2* gene lays at the 3′ end of *ZsMTL***a** locus whereas the gene at its 5′ end remained unknown. Because the position of *SLA2* gene on the right side of *MAT* is conserved in a number of pre-WGD (Butler *et al.* 2004; Gordon *et al.* 2011), we considered the *ZsMTL***a** locus as *MAT***a** expression locus. This hypothesis is supported by observing that in *S. cerevisiae* diploid cells, active MAT**a**1-MATα2 repressor is necessary to turn off the transcription for a set of haploid-specific genes. As being a diploid strain (Solieri *et al.* 2008), ABT301^T^ should express MAT**a**1 with the same functional role. Our preliminary expression analysis indicates that both *MAT***a**1 and *MAT*α2 are transcribed in ABT301^T^ strain in standard as well as salt stressed conditions, excluding that the *ZsMTL***a** locus is a silent cassette *HMR* (data not shown).

**Figure 7:**
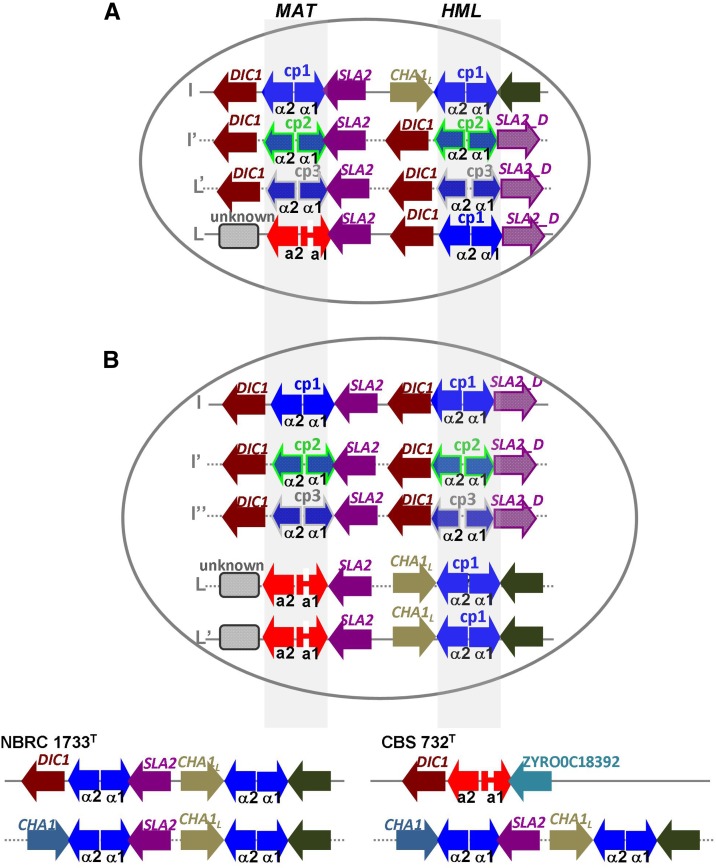
Inferred genomic organization of the three *ZsMTL*α copies (cp1, cp2, cp3) and one *ZsMTL*a in *Z. sapae* ABT301^T^. (A) First hypothesis considers a diploid genome with two sex chromosome pairs, namely I and I’ and L and L’, bearing *ZsMAT*α cassettes 1, 2, and 3, and *ZsMAT*a cassette, which are linked to the putative silent *ZsHML*α_D cassettes 1, 2, and 3 and to *ZsHML* copy 1 (cp1), respectively. (B) Second hypothesis considers an aneuploid number of chromosomes I. A set of three homeologous chromosomes, namely I, I’, and I”, harbor *ZsMAT*α cassettes 1, 2, and 3, arranged with *ZsHML*α_*D* cassettes 1, 2, and 3, respectively. Chromosomes L and L’ are homozygous for the *ZsMAT*a locus, which is linked to a silent *ZsHML* copy 1 cassette. In both chromosomal arrangements, *ZsHO* copies 1 and 2 are located on chromosome set I. Chromosomal letters are according to Figure S2. Dotted arrows indicate divergent *ZsMTL* sequences from *Z. rouxii*. Chromosomal organization of three cassette system in *Z. rouxii* haploid strains NBRC 1733^T^ and CBS 732^T^ were reported at the bottom for comparative purposes, according to Watanabe *et al.* (2013) and Souciet *et al.* (2009), respectively.

To explain the peculiar genetic makeup of *Z. sapae* mating system, we inferred two nonexclusive scenarios of chromosomal arrangement (Figure 7), considering two assumptions: (i) *MAT* and *HML* loci are linked in hemiascomycetes (Gordon *et al.* 2011); and (ii) *HMR* and/or *MAT* loci are located on different chromosomes in *Zygosaccharomyces* species (Fabre *et al.* 2005; Souciet *et al.* 2009; Watanabe *et al.* 2013). Based on the first scenario, diploid ABT301^T^ genome bears two genetically distinct sets of sex chromosome pairs, both lacking *HMR* cassettes. One set contains *MAT***a** and *MAT*α *Z. rouxii*-like sequences linked to *ZsHML_D* copy 1 and *ZsHML* copy 1, respectively. The other chromosome pair includes two slight divergent mating-type *α* loci, namely *ZsMAT*α copies 2 and 3, linked with the homologous *ZsHML*_*D* copies 2 and 3, respectively (Figure 7A). In the second scenario, the diploid ABT301^T^ strain has an aα genotype, homozygous for the *MAT***a**-*HML* loci (*ZsMAT***a**-*HML_D* copy 1) and heterozygous for the *MAT*α-*HML* loci (*ZsMAT*α copy 1-*ZsHML* copy 1 and Zs*MAT*α copy 2-*ZsHML* copy 2, respectively). Furthermore, consistently with this model, ABT301^T^ strain displays an homeologous extra-copy of sex chromosome (trisomy) which hosts the most divergent cassettes *ZsMAT*α copy 3-*ZsHML_D* copy 3 (Figure 7B). This hypothesis implies that ABT301^T^ is not an euploid strain with a karyotype that is a multiple of the haploid complement, a status which partially disagrees with our previous data (Solieri *et al.* 2008). By combining FACS and PFGE, strain ABT301^T^ and its conspecific ABT601 resulted to be diploid yeasts bearing additional number of chromosomes compared to *Z. rouxii*. Unfortunately, loss or gain of individual chromosomes similar in size would be hardly detectable even by combining FACS and PFGE. Therefore, the occurrence of an additional sex chromosome hosting syntenic array of *ZsMAT*α copy 3- *HML_D* copy 3 could be not excluded. However, in both scenarios the lack of *HMR* cassette implies that ABT301^T^ may be unable to reproduce by haplo-selfing. The loss of *HMR* cassette has been previously documented in *S. cerevisiae* haploid cells, where mutation or deletion of the *MAT*α locus on chromosome III causes reversion to the default *MAT***a** mating-type, allowing these *MAT*α cells, termed a-like fakers, to mate illegitimately with strains of the *MAT*α mating-type (Strathern *et al.* 1982). This event involves mitotic crossover at a frequency of 3.1 × 10^−6^ (Hiraoka *et al.* 2000), leading to a deletion between *MAT* and *HMR* or a circular chromosome fusing *MAT* and *HML* (Hawthorne 1963; Strathern *et al.* 1979; Haber *et al.* 1980). α,α homozygous diploid strains have been found via same-mating sex in *Cryptococcus neoformans* (Lin *et al.* 2005) and via parasexual cycle in *C. albicans* (Magee and Magee 2000; Wu *et al.* 2005; Forche *et al.* 2008). Among species having the silent cassette system, α,α,α strains have been found in *C. glabrata* (Srikantha *et al.* 2003), whereas α,α,α and α,α,α,α strains in *Z. rouxii* (Watanabe *et al.* 2013). In *Z. sapae*, an interchromosomal recombination may lead to the loss of *HMR* and the subsequent translocation of *ZsHOs* to the same chromosomes harboring *ZsMTL*α loci, giving rise to a chromosomal configuration different from that of *Z. rouxii* CBS 732^T^ (Souciet *et al.* 2009). In ABT301^T^ the resulting aααα genotype is likely to produce a mating-type imbalance, which determines the clonality as the main mode of reproduction observed in *Z. sapae* (Solieri *et al.* 2013a).

Our work provides a first insight to understand how the mating-type system is arranged in *Z. sapae* diploid genome. A question much harder to be addressed concerns why the *Z. sapae* genome is provided with a redundant number of divergent *MTL*α loci. Our hypothesis is that, although the *MAT* loci are typically nonrecombining genomic regions (Idnurm 2011), sex chromosome is a hotspot for DSBs, translocation, and mutation in *Z. sapae*. As in the relative *Z. rouxii*, in *Z. sapae* mating-type information is shared between two unlinked chromosomes, and this could favor outbreeding instead of inbreeding (Fraser and Heitman 2003). Illegitimate recombination at these “hot spots” can be induced by the exposure of *Zygosaccharomyces* yeasts to environmental stresses such as high osmotic conditions. This hypothesis is consistent with the results recently reported for haploid *Z. rouxii* strains (Watanabe *et al.* 2013) and with many reports that correlate increased DSBs frequencies to the upsurge of mutation rate and genome instability due to errors in DNA synthesis or microhomology-mediated jumps to ectopic templates (Hicks *et al.* 2010). DSBs that occur in *MAT* switching could trigger chromosomal rearrangements. When two specific DSBs are introduced simultaneously on separate chromosomes, DSBs-repair occurs via homologous recombination (with or without crossingover) (reviewed by Haber 2006) and in the absence of homology via nonhomologous end joining (Yu and Gabriel 2004), with reciprocal translocations and interchromosomal rearrangements. We speculate that under stress conditions imprecise mating-type switching and homeologous recombination between sex chromosomes further enrich the range of genetic diversity in *Zygosaccharomyces* species.

Furthermore, in *S. cerevisiae* the *MAT*-bearing chromosome III was found to be the most unstable chromosome (Kumaran *et al.* 2013) in haploinsufficiency (De Clare *et al.* 2011). Kumaran *et al.* (2013) suggest that chromosome III aberrant segregation during meiosis is mainly due to fast evolving centromeric sequences to which *MAT* and *HML* loci are tightly associated. This chromosome instability causes karyotype variability, giving rise to aneuploid descendants with diverse phenotypes. Chromosomal rearrangements, as well as sex chromosome instability, may result in a divergent adaptation with reproductive isolation and speciation (Dettman *et al.* 2007). Consistently with this thesis, *S. cerevisiae* (Magwene *et al.* 2011) and *C. albicans* (Forche *et al.* 2011) increase the number of recombination events in response to stress (fitness-associated recombination; Hadany and Beker 2003) to promote the evolution of complex traits and accelerate the adaptive rate. The present study provides a methodologic approach and sequence information to carry out a large-scale screening of mating-type loci organization in *Z. sapae* and *Z. rouxii*. This screening will be instrumental to confirm the role of genome plasticity and sex chromosome instability in stress adaptation.

## Supplementary Material

Supporting Information
