# Supplementary material for: Sex-Determination System in the Diploid Yeast Zygosaccharomyces sapae
Source: G3 (Bethesda). 2014 Jun 1;4(6):1011–25. doi: 10.1534/g3.114.010405 (PMC4065246; doi:10.1534/g3.114.010405)
Supplement: Supporting Information [file supp_4_6_1011__index.html]

Supporting Information 

# Sex-Determination System in the Diploid Yeast *Zygosaccharomyces sapae*

## Supporting Information for Solieri *et al.*, 2014

**Files in this Data Supplement:**

- Supporting Information - Tables S1-S4 and Figures S1-S5 (PDF, 982 KB)
- Table S1 - Degenerate primers used in this study. (PDF, 116 KB)
- Table S2 - List of gene-specific primers used for inverse PCR and PCR walking of *ZsMTL* loci and *HO* genes. (PDF, 148 KB)
- Table S3 - List of primers used for cassette system determination. (PDF, 116 KB)
- Table S4 - Restriction enzymes and primers for probe synthesis used in gDNA and PFGE-Southern blotting analyses. (PDF, 114 KB)
- Figure S1 - Outline of strategy used in *Zygosaccharomyces sapae HO* genes cloning. (PDF, 211 KB)
- Figure S2 - Chromosomal mapping of *ZsMTL*α, *ZsMTL***a** and *ZsHO* loci. (PDF, 136 KB)
- Figure S3 - Southern blot analysis of mating type cassettes and *HO* genes in *Zygoaccharomyces sapae* ABT301T. (PDF, 223 KB)
- Figure S4 - Z regions sequence comparisons from *Zygosaccharomyces sapae* strain ABT301T and *Zygosaccharomyces rouxii* CBS 732T. (PDF, 410 KB)
- Figure S5 - X regions sequence comparisons from *Zygosaccharomyces sapae* strain ABT301T and *Zygosaccharomyces rouxii* CBS 732T. (PDF, 397 KB)
