# Supplementary material for: Sex-Determination System in the Diploid Yeast Zygosaccharomyces sapae
Source: G3 (Bethesda). 2014 Jun 1;4(6):1011–25. doi: 10.1534/g3.114.010405 (PMC4065246; doi:10.1534/g3.114.010405)
Supplement: Supporting Information [file supp_4.6.1011_010405SI.pdf]

G3·Genes | Genomes | Genetics

Sex-determination system in the diploid yeast *Zygosaccharomyces sapae*

Lisa Solieri[1], Tikam Chand Dakal, Paolo Giudici, and Stefano Cassanelli

Department of Life Sciences, University of Modena and Reggio Emilia, via Amendola 2, Besta Building, 42122, Reggio Emilia, Italy.

[1]Corresponding author: Lisa Solieri, Department of Life Sciences, University of Modena and Reggio Emilia, via Amendola 2, Besta Building, 42122, Reggio Emilia, Italy. E-mail: lisa.solieri@unimore.it; Phone +39 0522 522057; Fax. +39 0522 522027.

Sequence data from this article have been deposited in the EMBL database under the accession numbers from HG931712 to HG931721.

DOI: 10.1534/g3.114.010405

Table S1 Degenerate primers used in this study

Protein	Domain* (N-C-terminal ends)	Primer	Sequence (5'-3')	PCR strategy	Plasmid	Insert length (bp)
MAT**a**1	85MRNCKG90	Zr-MATA1F1	ATG MGi AAY TGY AAR GGN AA	Zr-MATA1F1/Zr-MATA1R1	pA12	153
	129PIQVRIW135	Zr-MATA1R1	CCADATNCKiACYTGDATNGG			
MATα1	131NSFMAF136	Zr-MATalpha1-F2	AAY WSi TTY ATG GCN TTY	Zr-MATalpha1-F2/Zr_MATalpha1_R1 followed by Zr-MATalpha1-F2/Zr-MATalpha1-R2	pAlpha1.6	495
	170WDTFAQQFN178	Zr_MATalpha1_R1	RAAYTGYTGNGCRAANGTRTCCCA			
	184CGFVEWV190	Zr-MATalpha1-R2	CCCAYTCNACRAANCCRCA			
MATα2	1MNKIPIE7	Zr_MATALPHA2_F1	ATGAAYAARATHCCNATHGAR	Zr_MATALPHA2_F1/Zr_MATALPHA2_R1	pAlpha2.2	578
	187PQIKNWV193	Zr_MATALPHA2_R1	ACCCARTTYTTDATYTGNGG		pAlpha2.8	578
HO	55KHRAFEGE62	ZrHO_F2	AARCAYMGNGCNTTYGARGGNGA	ZrHO_F2/ZrHO_R2	pHO2.3	258
	133DFPMTPEG140	ZrHO_R2	ACCYTCNGGNGTCATNGGRAARTC		pHO2.8	258
	284LRKNNPFW292	ZrHO_F3	YTNMGNAARAAYAAYCCiTTYTGG	ZrHO_F3/ZrHO_R3	pHO3.5	147
	324FLAGLIDSD332	ZrHO_R3	TCNSWRTCDATNARNCCNGCiARRAA			

*Domain positions are referred to *Z. rouxii* proteins MAT**a**1 (GenBank: XP_002496431), MATα1 (GenBank: XP_002497889), MATα2 (GenBank: XP_002497888) and HO (GenBank: XP_2496098).

Table S2 List of gene-specific primers used for inverse PCR and PCR walking of *ZsMTL* loci and *HO* genes

Target	Primer code	Sequence (5'-3')	Description
*ZsMTL*α	301_MATa1F1	CCAAGAACTCTCGAAGAAGCTG	Primer specific for plasmid pAlpha1.6 and used to extend MATα1-like coding sequence,by *Hha*I-iPCR
	301_MATa1R1	GGCGGTGATGGAATCTTAGT	Primer specific for plasmid pAlpha1.6 and used to extend MATα1-like coding sequence,by *Hha*I-iPCR
	301_MATa1F2	GTTCGGAGAAGCCACTCAATTC	Primer specific for plasmid pAlpha1.6 and used to extend MATα1-like coding sequence,by *Hha*I-iPCR
	301_MATa1R2	TCATCCGCTATACACTTCCC	Primer specific for plasmid pAlpha1.6 and used to extend MATα1-like coding sequence by *Hha*I-iPCR
	301_MATa2F4	ATGGAGACTAAGTTATCGGGACC	Primer specific for plasmid pAlpha2.2 and used to extend MATα2-like coding sequence by *Hha*I-iPCR
	301_MATa2R4	CTCTTGATGTACTGGGTTGAGC	Primer specific for plasmid pAlpha2.2 and used to extend MATα2-like coding sequence by *Hha*I-iPCR
	301_MATa2F2	GAGCATCCTTACCTGCAAAC	Primer specific for plasmid pAlpha2.8 and used to extend MATα2-like coding sequence by *Hha*I-iPCR
	301_MATa2R2	GGAAGTATCTTCTCTAGCTCTGC	Primer specific for plasmid pAlpha2.8 and used to extend MATα2-like coding sequence by *Hha*I-iPCR
*ZsMTL***a**	301_MATA1F1	GGGAAGCTGTAGCAGCTAAT	Primer specific for plasmid pA12 and used to extend MATa1-like coding sequence by *Msp*I-iPCR
	301_MATA1R1	GTCCTCTTTCTCTCAAATACACG	Primer specific for plasmid pA12 and used to extend MATa1-like coding sequence by *Msp*I-iPCR
	301_MATA1F2	GCTGTAGCAGCTAATTGTGG	Primer specific for plasmid pA12 and used to extend MATa1-like coding sequence by *Msp*I-iPCR
	301_MATA1R2	CTCTTTCTCTCAAATACACGTTC	Primer specific for plasmid pA12 and used to extend MATa1-like coding sequence by *Msp*I-iPCR
	301_MATA2F1	GCAACATGGTCATGGTCAAC	Primer targeting 5' UTR of *Z. rouxii* CBS 732 *MAT***a**2 gene (ZYRO0C18326g) used for PCR-based 5' walking of *ZsMTL*a locus
	301_MATA2R1	TGAAGAGCACTGGCATCTAAA	*MAT***a**1-specific primer used in combination to 301_MATA2F1 for PCR-based 5' walking of *ZsMTLa* locus
HO	301_5'HOF1	CTACGTCGAGAGATCCATCATAG	primer specific for plasmid pHO2.3 used in combination with 301_5'HOR1
	301_5'HOF3	TCAGTGGCACATCAGCTT	primer specific for plasmid pHO2.8 used in combination with 301_5'HOR1
	301_5'HOR1	GCTTCACGCACCTGTAAATC	primer specific for plasmid pHO3.5
	UpHOCBS732F2	ACGAGTGGTGGTGGGATAGACTTA	primer targeting 5' UTR of *Z. rouxii* CBS 732 *HO* gene (ZYRO0C10428g); used for 5' PCR walking
	301_verylikeHOR3	CGCGAATCTACCGGTACTATT	*HO* copy 1-specific primer used in combination to UpHOCBS732F2; used for 5' PCR walking
	301_likeHOR3	CTACAAACCTACCGGTGTTAGA	*HO* copy 2-specific primer used in combination to UpHOCBS732F2; used for 5' PCR walking
	ZrHO_R5	CCNSWCCARTCNCKRTARAARTA	degenerate primer targeting the domain FYRDWSG at the C-terminal of *Z. rouxii* HO; used for 3' PCR walking
	DownHOCBS732R1	TCACCAAGGCTATGTCTTCTCGCT	primer targeting 3' UTR of *Z. rouxii* CBS 732 *HO* gene(ZYRO0C10428g) (ZYRO0C10428g); used for 3' PCR walking
	301_very_likeHOF5	TGTGATGGACATCGCAGAAATCGC	*HO* copy 1-specific primer used in combination to ZrHO_R5; used for 3' PCR walking
	301_very_likeHOF7	TGCATGCGGTGATCATTGTAAGGC	*HO* copy 1-specific primer used in combination to DownHOCBS732R1; used for 3' PCR walking
	301_likeHOF5	GGACATCGTAGAAACCGCCATTTG	*HO* copy 2-specific primer used in combination to ZrHO_R5; used for 3' PCR walking
	301_likeHOF7	ATGTTGTGGGCGTAACAGTTG	*HO* copy 2-specific primer used in combination to DownHOCBS732R1; used for 3' PCR walking

Table S3 List of primers used for cassette system determination

PCR walking	Gene	Primer	Sequence (5'->3')	Description	Reference
5' PCR-walking	*MAT***a**2	rev-a	CTCTTTCTCTCAAATACACGTTC	*MAT***a**2-specific reverse primer	this study
	*MAT*α2	rev-αcp3	TTAGGAGATAAAGGTAAGAATAGG	*MAT*α2 copy 3-specific reverse primer	this study
		rev-αcp1	CTT GGT AAT ACA GGT AAA GAG GGT	*MAT*α2 copy 1-specific reverse primer	this study
		rev-αcp2	GACACATTGCATTCTGTTAAACGT	*MAT*α2 copy 2-specific reverse primer	this study
	CHA1	1	GCTACTCCCTCATTAGAACATGAAA	forward primer specific for *CHA1* gene in CBS 732^T genome	Watanabe *et al*. 2013
	DIC1	2	CGCATGATATGAAACGAAGATGCAA	forward primer specific for *DIC1* gene in CBS 732^T genome	Watanabe *et al*. 2013
	CHA1_L	3	TACTTACTGGATGAATCTTCTGTGA	forward primer specific for *CHA1* paralog (ZYRO0F18524g) located near to the silent *HML* cassette in CBS 732^T genome	Watanabe *et al*. 2013
3' PCR walking	*MAT***a**1	for-a	GTAGCTTCCACAAGGTCTTCAAGG	*MAT***a**1-specific forward primer	this study
	*MAT*α1	for-αcp3	CCGCCGAAGAATTTACTTAGAG	*MAT*α1 copy 3-specific forward primer	this study
		for-αcp1	TTCCTTCACCGCCAGAGGTTC	*MAT*α1 copy 1-specific forward primer	this study
		for-αcp2	TTCCTTCACCTCCGGAGAACC	*MAT*α1 copy 2-specific forward primer	this study
	SLA2	A	CCAGTTAGTGTGTTATCGATAAGTC	reverse primer specific for *SLA2* gene in CBS 732^T genome	Watanabe *et al*. 2013
		DownMATa1R1	TTYGARTTYTAYGCNGAYTG	reverse degenerate primer targeting FEFYADC conserved amino acid sequence of *Z. rouxii* CBS 732^T *SLA2* gene	this study
	ZYRO0C18392	B	TCTATTTCGTCCGTTTATCGTTGGT	reverse primer specific for locus ZYRO0C18392g in CBS 732^T genome	Watanabe *et al*. 2013
		B'	CAGAGACTAATAATGAGAGAAAAGC	reverse primer specific for locus ZYRO0C18392g in CBS 732^T genome at 5' end of primer B	Watanabe *et al*. 2013
	ZYRO0F18634	C	TCAGTACCAGAAGTGGTCTTTGAAA	reverse primer specific for locus ZYRO0F18634g in CBS 732^T genome	Watanabe *et al*. 2013

Table S4 Restriction enzymes and primers for probe synthesis used in gDNA and PFGE-Southern blotting analyses

Target	Primer name	Sequence (5'->3')	Probe length (bp)	Restriction enzymes
*ZsMAT*α1	301_MATa1F2	GTTCGGAGAAGCCACTCAATTC	329	*Eco*RI, *Eco*RV, *Bam*HI, *Hae*III, *Ban*I
	301_MATa1R3	GCTGGCACAAGCTTCTCAACTCTA		
*ZsMAT***a**1	301_MATA1F3	GTAGCTTCCACAAGGTCTTCAAGG	585	*Eco*RI, *Eco*RV, *Hind*III, *Pvu*I, *Pst*I
	301_MATA1R3	GTGTCCAATCTACTTGTCAGACCCA		
HO	301_5’HOF4	CGCTGAGGACATCGATGAAA	631	
	301_3’HOR2	TTCAAATTCACCACGCAGTTCC		*Ava*I, *Ban*II, *Sac*II, *Pst*I, *Hind*III, *Bam*HI

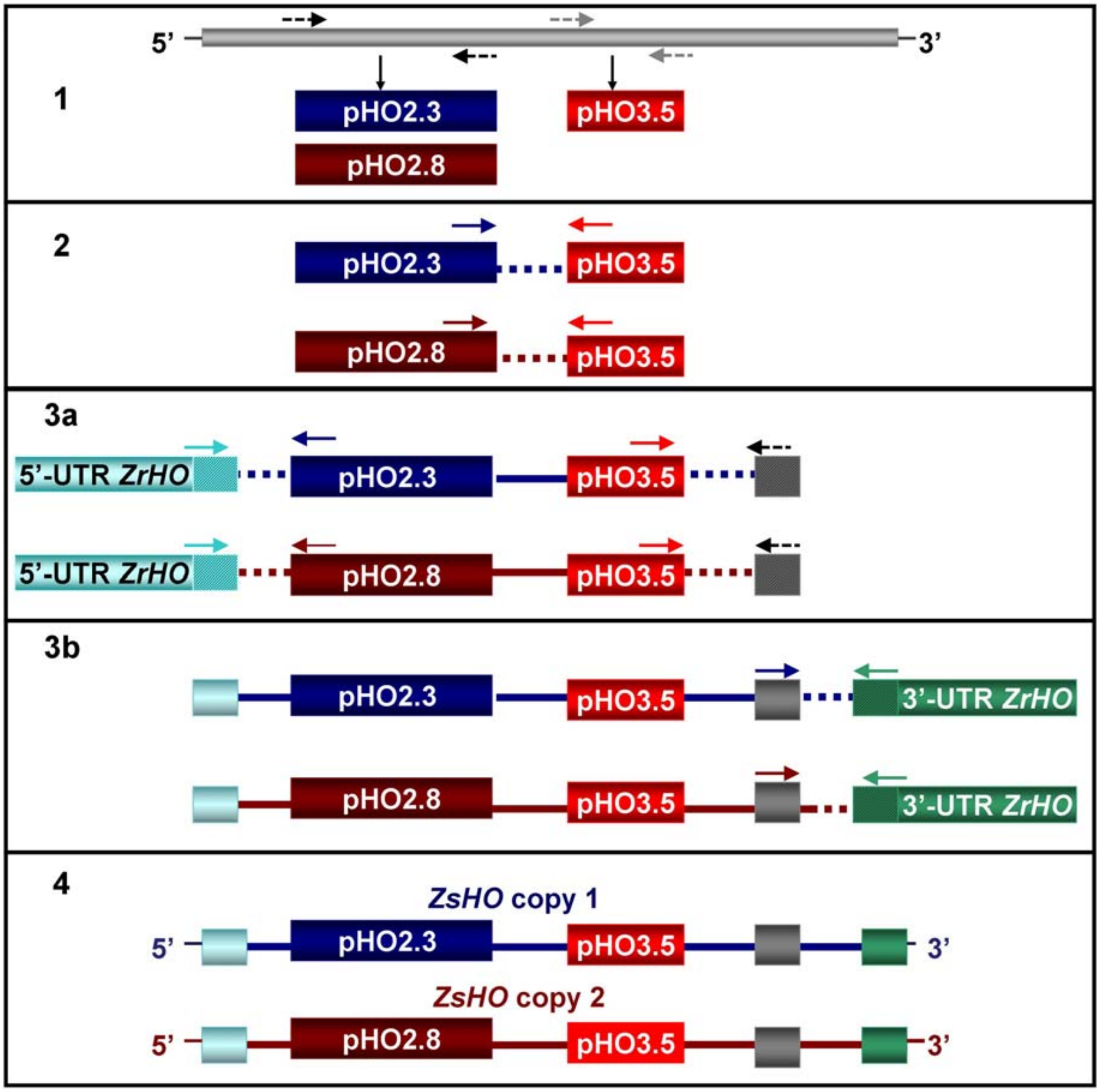

Figure S1 Outline of strategy used in *Zygosaccharomyces sapae HO* genes cloning. Numbers from 1 to 4 indicate the cloning and PCR walking steps. Dotted arrows represent degenerate primers and dotted lines undetermined sequences. Abbreviations: *ZrHO*, *Zygorsaccharomyces rouxii HO* gene; *ZsHO*, *Zygorsaccharomyces sapae HO* gene. Plasmid names according to Table S1.

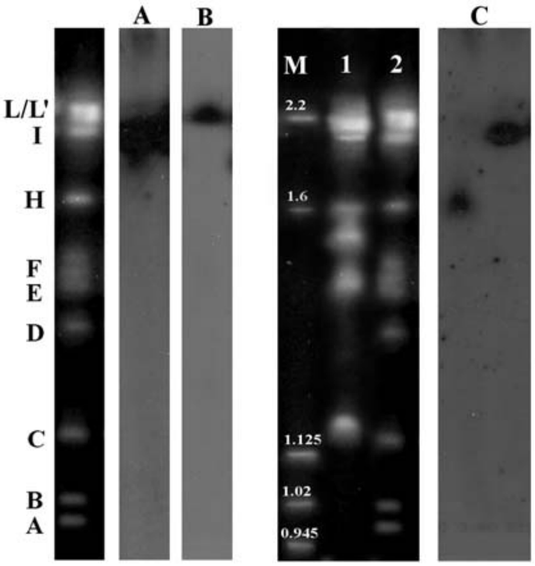

Figure S2 Chromosomal mapping of *ZsMTL*α, *ZsMTL***a** and *ZsHO* loci. Chromosomes were separated by PFGE for *Zygosaccharomyces rouxii* CBS 732^T (1) and *Zygosaccharomyces sapae* ABT301^T (2) and Southern blotting analyses were carried out with probes labeling to α-idiomorph loci (A), **a**-idiomorph locus (B), and *HO* genes (C), respectively. M indicates the chromosomal size ladder (*Saccharomyces cerevisiae* S288C, Bio-Rad Laboratories) is in megabase pairs (Mbp). *Z. sapae* chromosomes are indicated in uppercase letters.

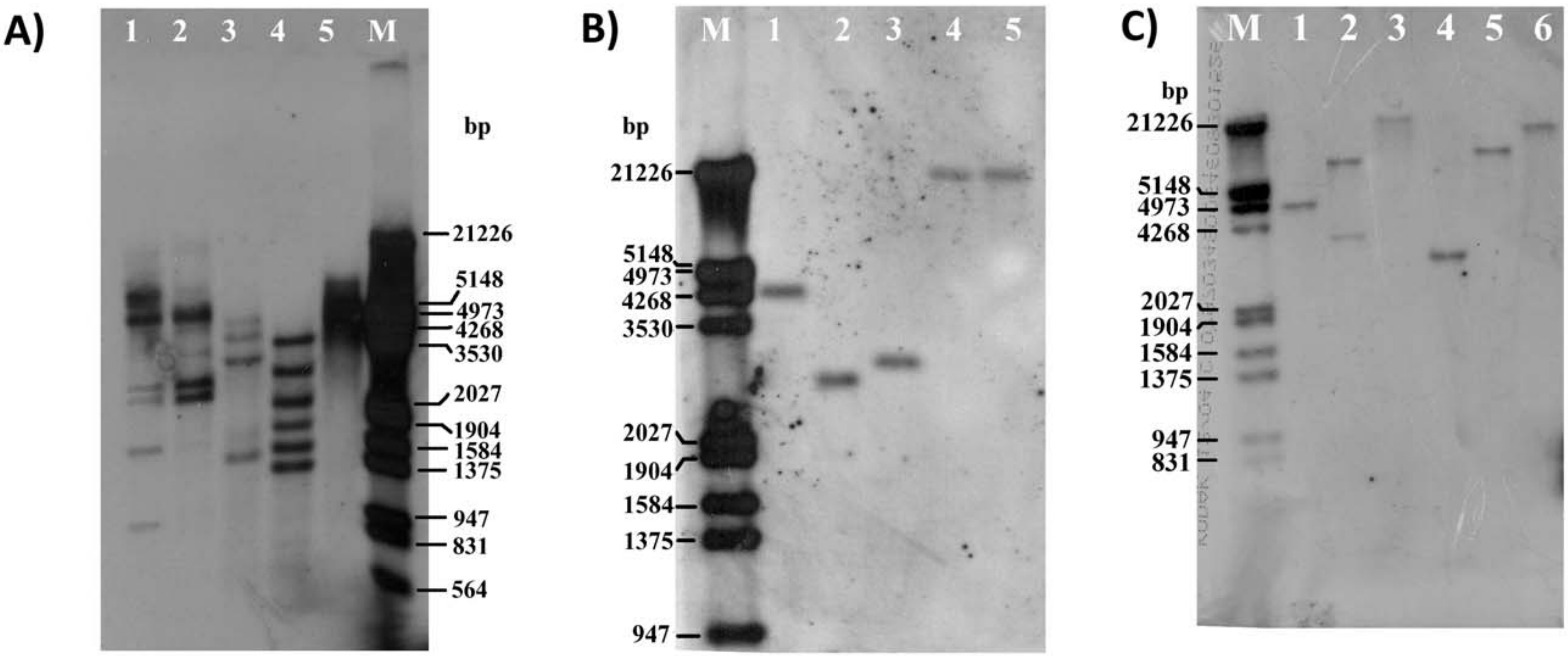

D)

Locus	Probe position (bp)*	Restriction enzyme sites					
		***Eco*RI**	***Eco*RV**	***Bam*HI**	***Hae*III**	***Ban*I**	
ZsMATα copy 1	from 1986 to 2314	2002	352	1894	847, 2309, 2408, 2520	-	
ZsMATα copy 2	from 2029 to 2357	-	399, 1340	1937	341, 2357, 2451, 2563	-	
ZsMATα copy 3	from 1990 to 2318	-	364	1898	306, 2313, 2412, 2524	-	
ZsHML_D copy 1	from 2005 to 2333	2021	317	1913	313, 866, 2836	-	
ZsHML_D copy 2	from 2001 to 2329	-	371, 1312	1909	2832	-	
ZsHML_D copy 3	from 1991 to 2319	2810	365	1899	307	-	
ZsHML copy 1	from 1987 to 2315	2003	353	1895	295, 848, 2310	-	
		***Eco*RI**	***Eco*RV**	***Hind*III**	***Pst*I**	***Pvu*I**	
*ZsMAT***a**	from 1255 to 1839	-	380	2013	-	-	
		***Ava*I**	***Ban*II**	***Sac*II**	***Pst*I**	***Hind*III**	***Bam*HI**
ZsHO copy 1	from 427 to 1059	39	31	-	-	-	-
ZsHO copy 2	from 374 to 1006	-	-	-	229	-	-

*Probe position and restriction site are according to sequences deposited in EMBL database (accession numbers from HG931712 to HG931721).

Figure S3 Southern blot analysis of mating type cassettes and *HO* genes in *Zygoaccharomyces sapae* ABT301^{T}. A) Genomic DNA was digested with *Eco*RI (1), *Eco*RV (2), *Bam*HI (3), *Hae*III (4), and *Ban*I (5) and analyzed with *ZsMTL*α probe in A), with *Eco*RI (1), *Eco*RV (2), *Hind*III (3), *Pst*I (4), and *Pvu*I (5), and analyzed with a *ZsMAT***a** probe in B); with *Ava*I (1), *Ban*II (2), *Sac*II (3), *Pst*I (4), *Hind*III (5), and *Bam*HI (6), and then analyzed with *HO* probe in C). In all plots the DNA molecular weight marker III (Roche) was used as DNA ladder (M). D) The table lists the restriction sites on the *Zygoaccharomyces sapae* mating type cassettes and *HO* genes using the indicated enzymes, and the positions of probes, according to sequences deposited under the accession numbers from HG931712 to HG931721.

```
                        HO-specific site
CBS 732                 CGCAGCAGTTTAATTTTGTCAAGCCGAAGTGTGGGTTTGTGGAGTGGGTG 50
ZsMATa                  CGCAGCAGTTTAATTTTGTCAAGCCGAAGTGTGGGTTTGTGGAGTGGGTG 50
ZsMATalpha copy 1       CGCAGCAGTTTAATTTTGTCAAGCCGAAGTGTGGGTTTGTGGAGTGGGTG 50
ZsHML copy 1            CGCAGCAGTTTAATTTTGTCAAGCCGAAGTGTGGGTTTGTGGAGTGGGTG 50
ZsMATalpha copy 2       CGCAGCAGTTTAATTTTGTCAAGCCGAAGTGTGGGTTTGTGGAGTGGGTG 50
ZsMATalpha copy 3       CGCAGCAGTTTAATTTTGTCAAGCCGAAGTGTGGGTTTGTGGAGTGGGTG 50
ZsHML_D copy 1          CGCAGCAGTTTAATTTTGTCAAGCCGAAGTGTGGGTTTGTGGAGTGGGTG 50
ZsHML_D copy 2          CGCAGCAGTTTAATTTTGTCAAGCCGAAGTGTGGGTTTGTGGAGTGGGTG 50
ZsHML_D copy 3          CGCAGCAGTTTAATTTTGTCAAGCCGAAGTGTGGGTTTGTGGAGTGGGTG 50
                        **************************************************

CBS 732                 GATCAAAGATATGAGCGGGAGAGTTGTATTTAGTTTGTTAAGAGTTGTTG 100
ZsMATa                  GATCAAAGATATGAGCGGGAGAGTTGTATTTAGTTTGTTAAGAGTTGTTG 100
ZsMATalpha copy 1       GATCAAAGATATGAGCGGGAGAGTTGTATTTAGTTTGTTAAGAGTTGTTG 100
ZsHML copy 1            GATCAAAGATATGAGCGGGAGAGTTGTATTTAGTTTGTTAAGAGTTGTTG 100
ZsMATalpha copy 2       GATCAAAGATATGAGCGGGAGAGTTGTATTTAGTTTGTTAAGAGTTGTTG 100
ZsMATalpha copy 3       GATCAAAGATATGAGCGGGAGAGTTGTATTTAGTTTGTTAAGAGTTGTTG 100
ZsHML_D copy 1          GATCAAAGATATGAGCGGGAGAGTTGTATTTAGTTTGTTAAGAGTTGTTG 100
ZsHML_D copy 2          GATCAAAGATATGAGCGGGAGAGTTGTATTTAGTTTGTTAAGAGTTGTTG 100
ZsHML_D copy 3          GATCAAAGATATGAGCGGGAGAGTTGTATTTAGTTTGTTAAGAGTTGTTG 100
                        **************************************************

CBS 732                 TAGATTTGTATTTGCATTGATGATGTCTTGTTGGGAGGGGGAAAGTAGTG 150
ZsMATa                  TAGATTTGTATTTGCATTGATGATGTCTTGTTGGGAGGGGGAAAGTAGTG 150
ZsMATalpha copy 1       TAGATTTGTATTTGCATTGATGATGTCTTGTTGGGAGGGGGAAAGTAGTG 150
ZsHML copy 1            TAGATTTGTATTTGCATTGATGATGTCTTGTTGGGAGGGGGAAAGTAGTG 150
ZsMATalpha copy 2       TAGATTTGTATTTGCATTGATGATGTCTTGTTGGGAGGGGGAAAGTAGTG 150
ZsMATalpha copy 3       TAGATTTGTATTTGCATTGATGATGTCTTGTTGGGAGGGGGAAAGTAGTG 150
ZsHML_D copy 1          TAGATTTGTATTTGCATTGATGATGTCTTGTTGGGAGGGGGAAAGTAGTG 150
ZsHML_D copy 2          TAGATTTGTATTTGCATTGATGATGTCTTGTTGGGAGGGGGAAAGTAGTG 150
ZsHML_D copy 3          TAGATTTGTATTTGCATTGATGATGTCTTGTTGGGAGGGGGAAAGTAGTG 150
                        **************************************************

CBS 732                 CTTGGAAGTAGAGTTGAGAAGCTTGGGCCAGCAGTGGCGATGGATTGTGT 200
ZsMATa                  CTTGGAAGTAGAGTTGAGAAGCTTGGGCCAGCAGTGGCGATGGATTGTGT 200
ZsMATalpha copy 1       CTTGGAAGTAGAGTTGAGAAGCTTGGGCCAGCAGTGGCGATGGATTGTGT 200
ZsHML copy 1            CTTGGAAGTAGAGTTGAGAAGCTTGGGCCAGCAGTGGCGATGGATTGTGT 200
ZsMATalpha copy 2       CTTGGAAGTAGAGTTGAGAAGCTTGGGCCAGCAGTGGCGATGGATTGTGT 200
ZsMATalpha copy 3       CTTGGAAGTAGAGTTGAGAAGCTTGGGCCAGCAGTGGCGATGGATTGTGT 200
ZsHML_D copy 1          CTTGGAAGTAGAGTTGAGAAGCTTGTGCCAGCACTGGCGATGGATTGTGT 200
ZsHML_D copy 2          CTTGGAAGTAGAGTTGAGAAGCTTGTGCCAGCACTGGCGATGGATTGTGT 200
ZsHML_D copy 3          CTTGGAAGTAGAGTTGAGAAGCTTGTGCCAGCACTGGCGATGGATTGTGT 200
                        ************************* ******* ****************

CBS 732                 TGTAGTAGTGTAGTTATCTGATGCGTTAATTGTATTATAGT 241
ZsMATa                  TGTAGTAGTGTAGTTATCTGATGCGTTAATTGTATTATAGT 241
ZsMATalpha copy 1       TGTAGTAGTGTAGTTATCTGATGCGTTAATTGTATTATAGT 241
ZsHML copy 1            TGTAGTAGTGTAGTTATCTGATGCGTTAATTGTATTATAGT 241
ZsMATalpha copy 2       TGTAGTAGTGTAGTTATCTGATGCGTTAATTGTATTATAGT 241
ZsMATalpha copy 3       TGTAGTAGTGTAGTTATCTGATGCGTTAATTGTATTATAGT 241
ZsHML_D copy 1          TGTAGTAGTGTACTGATCTGATGGGTTAATTGTATTAGGCT 241
ZsHML_D copy 2          TGTAGTAGTGTACTGATCTGATGGGTTAATTGTATTAGGCT 241
ZsHML_D copy 3          TGTAGTAGTGTACTGATCTGATGGGTTAATTGTATTAGGCT 241
                        ************ * ******** *************  . *
```

Figure S4 Z regions sequence comparisons from *Zygosaccharomyces sapae* strain ABT301^T and *Zygosaccharomyces rouxii* CBS 732^T. Aligned Z sequences of eight *Z. sapae* (Zs) mating type cassettes: *ZsMAT*α copies 1, 2, 3, *ZsHML_D* copies 1, 2, and 3, *ZsHML* copy 1, and *ZsMAT***a**.

```
CBS 732                AGCGATTTGCTGGACGGCGGAGGCGGGGCGGCGGAGGCGGGGCGGGGGCG 50
ZsHML copy 1           AGCGATTTGCTGGACGGCGGAGGCGGGGCGGCGGAGGCGGGGCGGGGGCG 50
ZsMATalpha copy 1      AGCGATTTGCTGGACGGCGGAGGCGGGGCGGCGTAGGCGGGGCGGGGGCG 50
ZsMATa                 AGCGATTTGCTGGACGGCGGAGGCGGGGCGGCGTAGGCGGGGCGGGGGCG 50
ZsMATalpha copy 2      AGCGATTTGCTGGACGGCGGAGGCGGGGCGGCGTAGGCGGGGCGGGGGCG 50
ZsHML_D copy 1         AGCGATTTGCTGGACGGCGGAGGCGGGGCGGCGTAGGCGGGGCGGGGGCG 50
ZsHML_D copy 2         AGCGATTTGCTGGACGGCGGAGGCGGGGCGGCGTAGGCGGGGCGGGGGCG 50
ZsMATalpha copy 3      AGCGATTTGCTGGACGGCGGAGGCGGGGCGGCGTAGGCGGGGCGGGGGCG 50
ZsHML_D copy 3         AGCGATTTGCTGGACGGCGGAGGCGGGGCGGCGTAGGCGGGGCGGGGGCG 50
                       ********************************* ****************

CBS 732                ATGGTTTTTTCTTGGGGTGGATTCGCTGCTTGGAGACTTTGCCGCCGGGG 100
ZsHML copy 1           ATGGTTTTTTCTTGGGGTGGATTCGCTGCTTGGAGACTTTGCCGCCGGGG 100
ZsMATalpha copy 1      ATGGTTTTTTCTTGGGGTGGATTCGCTGCTTGGAGACTTTGCCGCCGGGG 100
ZsMATa                 ATGGTTTTTTCTTGGGGTGGATTCGCTGCTTGGAGACTTTGCCGCCGGGG 100
ZsMATalpha copy 2      ATGGTTTTTTCTTGGGGTGGATTCGCTGCTTGGAGACTTTGCCGCCGGGG 100
ZsHML_D copy 1         ATGGTTTTTTCTTGGGGTGGATTCGCTGCTTGGAGACTTTGCCGCCGGGG 100
ZsHML_D copy 2         ATGGTTTTTTCTTGGGGTGGATTCGCTGCTTGGAGACTTTGCCGCCGGGG 100
ZsMATalpha copy 3      ATGGTTTTTTCTTGGGGTGGATTCGCTGCTTGGAGACTTTGCCGCCGGGG 100
ZsHML_D copy 3         ATGGTTTTTTCTTGGGGTGGATTCGCTGCTTGGAGACTTTGCCGCCGGGG 100
                       **************************************************

CBS 732                GCGGGTTTTGTTTTTGCATTTCTCTCAGCGTTGGTTGCAGGTGGGCTGG 150
ZsHML copy 1           GCGGGTTTTGTTTTTGCATTTCTCTCAGCGTTGGTTGCAGGTGGGCTGG 150
ZsMATalpha copy 1      GCGGGTTTTGTTTTTGCATTTCTCTCAGCGTTGGTTGCAGGTGGGCTGG 150
ZsMATa                 GCGGGTTTTGTTTTTGCATTTCTCTCAGCGTTGGTTGCAGGTGGGCTGG 150
ZsMATalpha copy 2      GCGGGTTTTGTTTTTGCATTTCTCTCAGCGTTGGTTGCAGGTGGGCTGG 150
ZsHML_D copy 1         GCGGGTTTTGTTTTTGCATTTCTCTCAGCGTTGGTTGCAGGTGGGCTGG 150
ZsHML_D copy 2         GCGGGTTTTGTTTTTGCATTTCTCTCAGCGTTGGTTGCAGGTGGGCTGG 150
ZsMATalpha copy 3      GCGGGTTTTGTTTTTGCATTTCTCTCAGCGTTGGTTGCAGGTGGGCTGG 150
ZsHML_D copy 3         GCGGGTTTTGTTTTTGCATTTCTCTCAGCGTTGGTTGCAGGTGGGCTGG 150
                       **************************************************

CBS 732                GGCGGCAGGGTTGGCGGCTTGGGCGTTGGCGGCTTGTGCTTGTGCTTGTG 200
ZsHML copy 1           GGCGGCAGGGTTGGCGGCTTGGGCGTTGGCGGCTTGTGCTTGTGCTTGTG 200
ZsMATalpha copy 1      GGCGGCAGGGTTGGCGGCTTG---------------------------TG 173
ZsMATa                 GGCGGCAGGGTTGGCGGCTTG---------------------------TG 173
ZsMATalpha copy 2      GGCGGCAGGGTTGGCGGCTTG---------------------------TG 173
ZsHML_D copy 1         GGCGGCAGGGTTGGCGGCTTG---------------------------TG 173
ZsHML_D copy 2         GGCGGCAGGGTTGGCGGCTTG---------------------------TG 173
ZsMATalpha copy 3      GGCGGCAGGGTTGGCGGCTTG---------------------------TG 173
ZsHML_D copy 3         GGCGGCAGGGTTGGCGGCTTG---------------------------TG 173
                       *********************                           **

CBS 732                CTTGTGCTTGCGCTTTGGCGGCGGCGGCGGCGGCAGGACGCCGCAGG 250
ZsHML copy 1           CTTGTGCTTGCGCTTTGGCGGCGGCGGCGGCGGCAGGACGCCGCAGG 250
ZsMATalpha copy 1      CTTGTGCTTGCGCTTTGGCGGCGGCGGCGGCGGCAGGACGCCGCAGG 223
ZsMATa                 CTTGTGCTTGCGCTTTGGCGGCGGCGGCGGCGGCAGGACGCCGCAGG 223
ZsMATalpha copy 2      CTTGTGCTTGCGCTTTGGCGGCGGCGGCGGCGGCAGGACGCCGCAGG 223
ZsHML_D copy 1         CTTGTGCTTGCGCTTTGGCGGCGGCGGCGGCGGCAGGACGCCGCAGG 223
ZsHML_D copy 2         CTTGTGCTTGCGCTTTGGCGGCGGCGGCGGCGGCAGGACGCCGCAGG 223
ZsMATalpha copy 3      CTTGTGCTTGCGCTTTGGCGGCGGCGGCGGCGGCAGGACGCCGCAGG 223
ZsHML_D copy 3         CTTGTGCTTGCGCTTTGGCGGCGGCGGCGGCGGCAGGACGCCGCAGG 223
                       **************************************************
```

Figure S5 X regions sequence comparisons from *Zygosaccharomyces sapae* strain ABT301[T] and *Zygosaccharomyces rouxii* CBS 732[T]. Partial aligned X sequences of eight *Z. sapae* (Zs) mating type cassettes: *ZsMAT*α copies 1, 2, 3, *ZsHML_D* copies 1, 2, and 3, *ZsHML* copy 1, and *ZsMAT***a**.
