# Supplementary material for: Sex-Determination System in the Diploid Yeast Zygosaccharomyces sapae
Source: G3 (Bethesda). 2014 Jun 1;4(6):1011–25. doi: 10.1534/g3.114.010405 (PMC4065246; doi:10.1534/g3.114.010405)
Supplement: Supporting Information [file supp_4.6.1011_TableS1.pdf]

Table S1 Degenerate primers used in this study

Protein	Domain* (N-C-terminal ends)	Primer	Sequence (5'-3')	PCR strategy	Plasmid	Insert length (bp)
MAT**a**1	85MRNCKG90	Zr-MATA1F1	ATG MGi AAY TGY AAR GGN AA	Zr-MATA1F1/Zr-MATA1R1	pA12	153
	129PIQVRIW135	Zr-MATA1R1	CCADATNCKiACYTGDATNGG			
MATα1	131NSFMAF136	Zr-MATalpha1-F2	AAY WSi TTY ATG GCN TTY	Zr-MATalpha1-F2/Zr_MATalpha1_R1 followed by Zr-MATalpha1-F2/Zr-MATalpha1-R2	pAlpha1.6	495
	170WDTFAQQFN178	Zr_MATalpha1_R1	RAAYTGYTGNGCRAANGTRTCCCA			
	184CGFVEWV190	Zr-MATalpha1-R2	CCCAYTCNACRAANCCRCA			
MATα2	1MNKIPIE7	Zr_MATALPHA2_F1	ATGAAYAARATHCCNATHGAR	Zr_MATALPHA2_F1/Zr_MATALPHA2_R1	pAlpha2.2	578
	187PQIKNWV193	Zr_MATALPHA2_R1	ACCCARTTYTTDATYTGNGG		pAlpha2.8	578
HO	55KHRAFEGE62	ZrHO_F2	AARCAYMGNGCNTTYGARGGNGA	ZrHO_F2/ZrHO_R2	pHO2.3	258
	133DFPMTPEG140	ZrHO_R2	ACCYTCNGGNGTCATNGGRAARTC		pHO2.8	258
	284LRKNNPFW292	ZrHO_F3	YTNMGNAARAAYAAYCCiTTYTGG	ZrHO_F3/ZrHO_R3	pHO3.5	147
	324FLAGLIDSD332	ZrHO_R3	TCNSWRTCDATNARNCCNGCiARRAA			

*Domain positions are referred to *Z. rouxii* proteins MAT**a**1 (GenBank: XP_002496431), MATα1 (GenBank: XP_002497889), MATα2 (GenBank: XP_002497888) and HO (GenBank: XP_2496098).
