# Supplementary material for: Sex-Determination System in the Diploid Yeast Zygosaccharomyces sapae
Source: G3 (Bethesda). 2014 Jun 1;4(6):1011–25. doi: 10.1534/g3.114.010405 (PMC4065246; doi:10.1534/g3.114.010405)
Supplement: Supporting Information [file supp_4.6.1011_TableS2.pdf]

Table S2 List of gene-specific primers used for inverse PCR and PCR walking of *ZsMTL* loci and *HO* genes

Target	Primer code	Sequence (5'-3')	Description
*ZsMTL*α	301_MATa1F1	CCAAGAACTCTCGAAGAAGCTG	Primer specific for plasmid pAlpha1.6 and used to extend MATα1-like coding sequence,by *Hha*I-iPCR
	301_MATa1R1	GGCGGTGATGGAATCTTAGT	Primer specific for plasmid pAlpha1.6 and used to extend MATα1-like coding sequence,by *Hha*I-iPCR
	301_MATa1F2	GTTCGGAGAAGCCACTCAATTC	Primer specific for plasmid pAlpha1.6 and used to extend MATα1-like coding sequence,by *Hha*I-iPCR
	301_MATa1R2	TCATCCGCTATACACTTCCC	Primer specific for plasmid pAlpha1.6 and used to extend MATα1-like coding sequence by *Hha*I-iPCR
	301_MATa2F4	ATGGAGACTAAGTTATCGGGACC	Primer specific for plasmid pAlpha2.2 and used to extend MATα2-like coding sequence by *Hha*I-iPCR
	301_MATa2R4	CTCTTGATGTACTGGGTTGAGC	Primer specific for plasmid pAlpha2.2 and used to extend MATα2-like coding sequence by *Hha*I-iPCR
	301_MATa2F2	GAGCATCCTTACCTGCAAAC	Primer specific for plasmid pAlpha2.8 and used to extend MATα2-like coding sequence by *Hha*I-iPCR
	301_MATa2R2	GGAAGTATCTTCTCTAGCTCTGC	Primer specific for plasmid pAlpha2.8 and used to extend MATα2-like coding sequence by *Hha*I-iPCR
*ZsMTL***a**	301_MATA1F1	GGGAAGCTGTAGCAGCTAAT	Primer specific for plasmid pA12 and used to extend MATa1-like coding sequence by *Msp*I-iPCR
	301_MATA1R1	GTCCTCTTTCTCTCAAATACACG	Primer specific for plasmid pA12 and used to extend MATa1-like coding sequence by *Msp*I-iPCR
	301_MATA1F2	GCTGTAGCAGCTAATTGTGG	Primer specific for plasmid pA12 and used to extend MATa1-like coding sequence by *Msp*I-iPCR
	301_MATA1R2	CTCTTTCTCTCAAATACACGTTC	Primer specific for plasmid pA12 and used to extend MATa1-like coding sequence by *Msp*I-iPCR
	301_MATA2F1	GCAACATGGTCATGGTCAAC	Primer targeting 5' UTR of *Z. rouxii* CBS 732 *MAT***a**2 gene (ZYRO0C18326g) used for PCR-based 5' walking of *ZsMTL*a locus
	301_MATA2R1	TGAAGAGCACTGGCATCTAAA	*MAT***a**1-specific primer used in combination to 301_MATA2F1 for PCR-based 5' walking of *ZsMTLa* locus
HO	301_5'HOF1	CTACGTCGAGAGATCCATCATAG	primer specific for plasmid pHO2.3 used in combination with 301_5'HOR1
	301_5'HOF3	TCAGTGGCACATCAGCTT	primer specific for plasmid pHO2.8 used in combination with 301_5'HOR1
	301_5'HOR1	GCTTCACGCACCTGTAAATC	primer specific for plasmid pHO3.5
	UpHOCBS732F2	ACGAGTGGTGGTGGGATAGACTTA	primer targeting 5' UTR of *Z. rouxii* CBS 732 *HO* gene (ZYRO0C10428g); used for 5' PCR walking
	301_verylikeHOR3	CGCGAATCTACCGGTACTATT	*HO* copy 1-specific primer used in combination to UpHOCBS732F2; used for 5' PCR walking
	301_likeHOR3	CTACAAACCTACCGGTGTTAGA	*HO* copy 2-specific primer used in combination to UpHOCBS732F2; used for 5' PCR walking
	ZrHO_R5	CCNSWCCARTCNCKRTARAARTA	degenerate primer targeting the domain FYRDWSG at the C-terminal of *Z. rouxii* HO; used for 3' PCR walking
	DownHOCBS732R1	TCACCAAGGCTATGTCTTCTCGCT	primer targeting 3' UTR of *Z. rouxii* CBS 732 *HO* gene(ZYRO0C10428g) (ZYRO0C10428g); used for 3' PCR walking
	301_very_likeHOF5	TGTGATGGACATCGCAGAAATCGC	*HO* copy 1-specific primer used in combination to ZrHO_R5; used for 3' PCR walking
	301_very_likeHOF7	TGCATGCGGTGATCATTGTAAGGC	*HO* copy 1-specific primer used in combination to DownHOCBS732R1; used for 3' PCR walking
	301_likeHOF5	GGACATCGTAGAAACCGCCATTTG	*HO* copy 2-specific primer used in combination to ZrHO_R5; used for 3' PCR walking
	301_likeHOF7	ATGTTGTGGGCGTAACAGTTG	*HO* copy 2-specific primer used in combination to DownHOCBS732R1; used for 3' PCR walking
