# Supplementary material for: Sex-Determination System in the Diploid Yeast Zygosaccharomyces sapae
Source: G3 (Bethesda). 2014 Jun 1;4(6):1011–25. doi: 10.1534/g3.114.010405 (PMC4065246; doi:10.1534/g3.114.010405)
Supplement: Supporting Information [file supp_4.6.1011_TableS3.pdf]

Table S3 List of primers used for cassette system determination

PCR walking	Gene	Primer	Sequence (5'->3')	Description	Reference
5' PCR-walking	*MAT***a**2	rev-a	CTCTTTCTCTCAAATACACGTTC	*MAT***a**2-specific reverse primer	this study
	*MAT*α2	rev-αcp3	TTAGGAGATAAAGGTAAGAATAGG	*MAT*α2 copy 3-specific reverse primer	this study
		rev-αcp1	CTT GGT AAT ACA GGT AAA GAG GGT	*MAT*α2 copy 1-specific reverse primer	this study
		rev-αcp2	GACACATTGCATTCTGTTAAACGT	*MAT*α2 copy 2-specific reverse primer	this study
	CHA1	1	GCTACTCCCTCATTAGAACATGAAA	forward primer specific for *CHA1* gene in CBS 732^T genome	Watanabe *et al*. 2013
	DIC1	2	CGCATGATATGAAACGAAGATGCAA	forward primer specific for *DIC1* gene in CBS 732^T genome	Watanabe *et al*. 2013
	CHA1_L	3	TACTTACTGGATGAATCTTCTGTGA	forward primer specific for *CHA1* paralog (ZYRO0F18524g) located near to the silent *HML* cassette in CBS 732^T genome	Watanabe *et al*. 2013
3' PCR walking	*MAT***a**1	for-a	GTAGCTTCCACAAGGTCTTCAAGG	*MAT***a**1-specific forward primer	this study
	*MAT*α1	for-αcp3	CCGCCGAAGAATTTACTTAGAG	*MAT*α1 copy 3-specific forward primer	this study
		for-αcp1	TTCCTTCACCGCCAGAGGTTC	*MAT*α1 copy 1-specific forward primer	this study
		for-αcp2	TTCCTTCACCTCCGGAGAACC	*MAT*α1 copy 2-specific forward primer	this study
	SLA2	A	CCAGTTAGTGTGTTATCGATAAGTC	reverse primer specific for *SLA2* gene in CBS 732^T genome	Watanabe *et al*. 2013
		DownMATa1R1	TTYGARTTYTAYGCNGAYTG	reverse degenerate primer targeting FEFYADC conserved amino acid sequence of *Z. rouxii* CBS 732^T *SLA2* gene	this study
	ZYRO0C18392	B	TCTATTTCGTCCGTTTATCGTTGGT	reverse primer specific for locus ZYRO0C18392g in CBS 732^T genome	Watanabe *et al*. 2013
		B'	CAGAGACTAATAATGAGAGAAAAGC	reverse primer specific for locus ZYRO0C18392g in CBS 732^T genome at 5' end of primer B	Watanabe *et al*. 2013
	ZYRO0F18634	C	TCAGTACCAGAAGTGGTCTTTGAAA	reverse primer specific for locus ZYRO0F18634g in CBS 732^T genome	Watanabe *et al*. 2013
