# Supplementary material for: Sex-Determination System in the Diploid Yeast Zygosaccharomyces sapae
Source: G3 (Bethesda). 2014 Jun 1;4(6):1011–25. doi: 10.1534/g3.114.010405 (PMC4065246; doi:10.1534/g3.114.010405)
Supplement: Supporting Information [file supp_4.6.1011_TableS4.pdf]

Table S4 Restriction enzymes and primers for probe synthesis used in gDNA and PFGE-Southern blotting analyses

Target	Primer name	Sequence (5'->3')	Probe length (bp)	Restriction enzymes
*ZsMAT*α1	301_MATa1F2	GTTCGGAGAAGCCACTCAATTC	329	*Eco*RI, *Eco*RV, *Bam*HI, *Hae*III, *Ban*I
	301_MATa1R3	GCTGGCACAAGCTTCTCAACTCTA		
*ZsMAT***a**1	301_MATA1F3	GTAGCTTCCACAAGGTCTTCAAGG	585	*Eco*RI, *Eco*RV, *Hind*III, *Pvu*I, *Pst*I
	301_MATA1R3	GTGTCCAATCTACTTGTCAGACCCA		
HO	301_5’HOF4	CGCTGAGGACATCGATGAAA	631	
	301_3’HOR2	TTCAAATTCACCACGCAGTTCC		*Ava*I, *Ban*II, *Sac*II, *Pst*I, *Hind*III, *Bam*HI
