# Supplementary material for: Sex-Determination System in the Diploid Yeast Zygosaccharomyces sapae
Source: G3 (Bethesda). 2014 Jun 1;4(6):1011–25. doi: 10.1534/g3.114.010405 (PMC4065246; doi:10.1534/g3.114.010405)
Supplement: Supporting Information [file supp_4.6.1011_FigureS1.pdf]

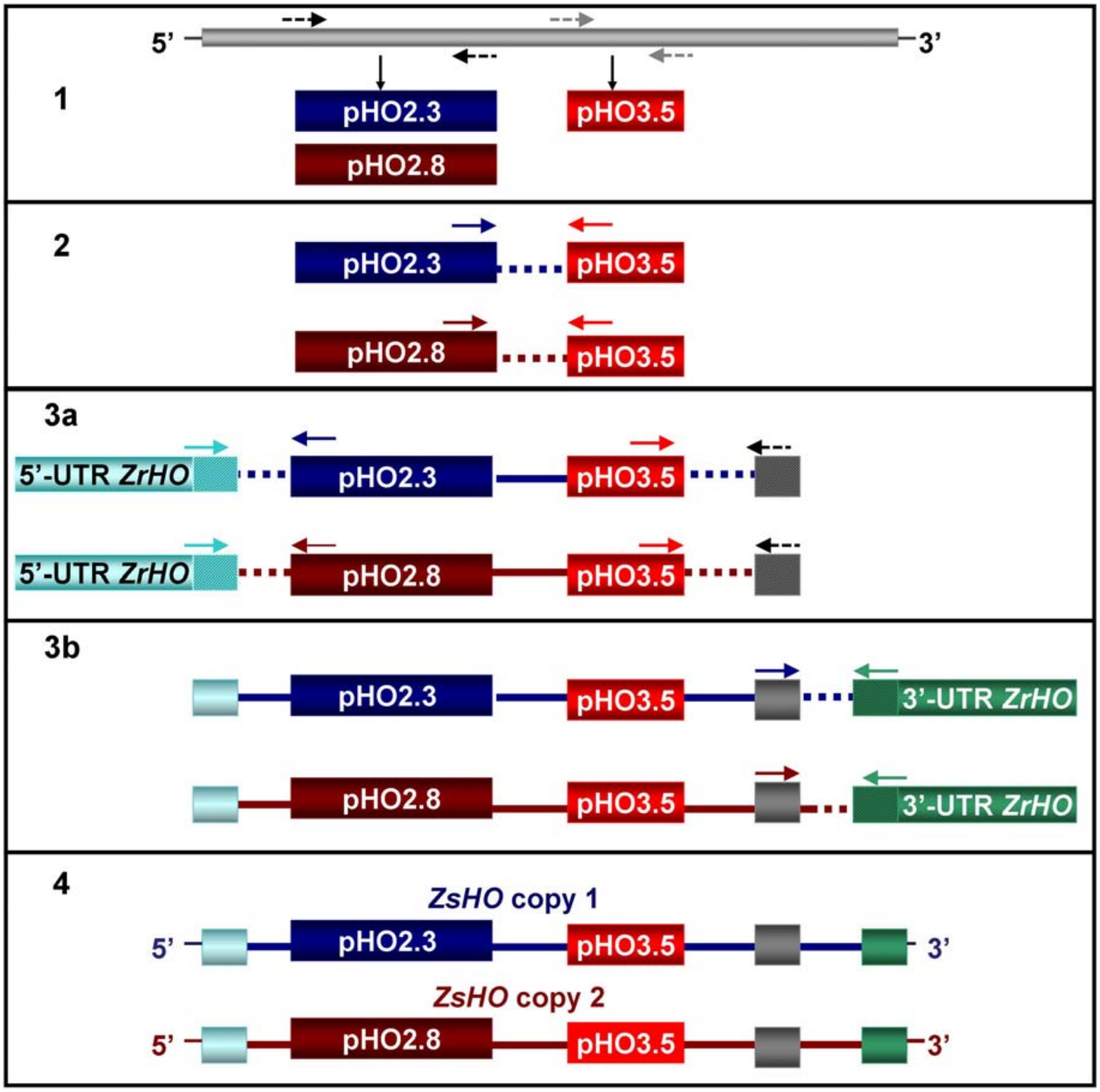

Figure S1 Outline of strategy used in *Zygosaccharomyces sapae HO* genes cloning. Numbers from 1 to 4 indicate the cloning and PCR walking steps. Dotted arrows represent degenerate primers and dotted lines undetermined sequences. Abbreviations: *ZrHO*, *Zygorsaccharomyces rouxii HO* gene; *ZsHO*, *Zygorsaccharomyces sapae HO* gene. Plasmid names according to Table S1.
