# Supplementary material for: Sex-Determination System in the Diploid Yeast Zygosaccharomyces sapae
Source: G3 (Bethesda). 2014 Jun 1;4(6):1011–25. doi: 10.1534/g3.114.010405 (PMC4065246; doi:10.1534/g3.114.010405)
Supplement: Supporting Information [file supp_4.6.1011_FigureS2.pdf]

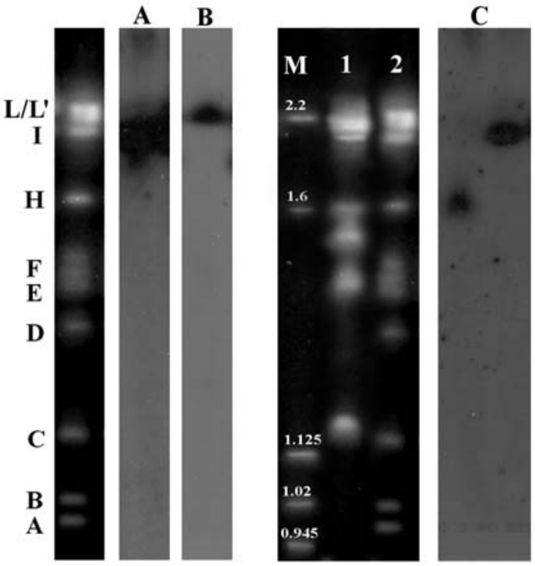

Figure S2 Chromosomal mapping of *ZsMTL*α, *ZsMTL***a** and *ZsHO* loci. Chromosomes were separated by PFGE for *Zygosaccharomyces rouxii* CBS 732^T (1) and *Zygosaccharomyces sapae* ABT301^T (2) and Southern blotting analyses were carried out with probes labeling to α-idiomorph loci (A), **a**-idiomorph locus (B), and *HO* genes (C), respectively. M indicates the chromosomal size ladder (*Saccharomyces cerevisiae* S288C, Bio-Rad Laboratories) is in megabase pairs (Mbp). *Z. sapae* chromosomes are indicated in uppercase letters.
