# Supplementary material for: Sex-Determination System in the Diploid Yeast Zygosaccharomyces sapae
Source: G3 (Bethesda). 2014 Jun 1;4(6):1011–25. doi: 10.1534/g3.114.010405 (PMC4065246; doi:10.1534/g3.114.010405)
Supplement: Supporting Information [file supp_4.6.1011_FigureS3.pdf]

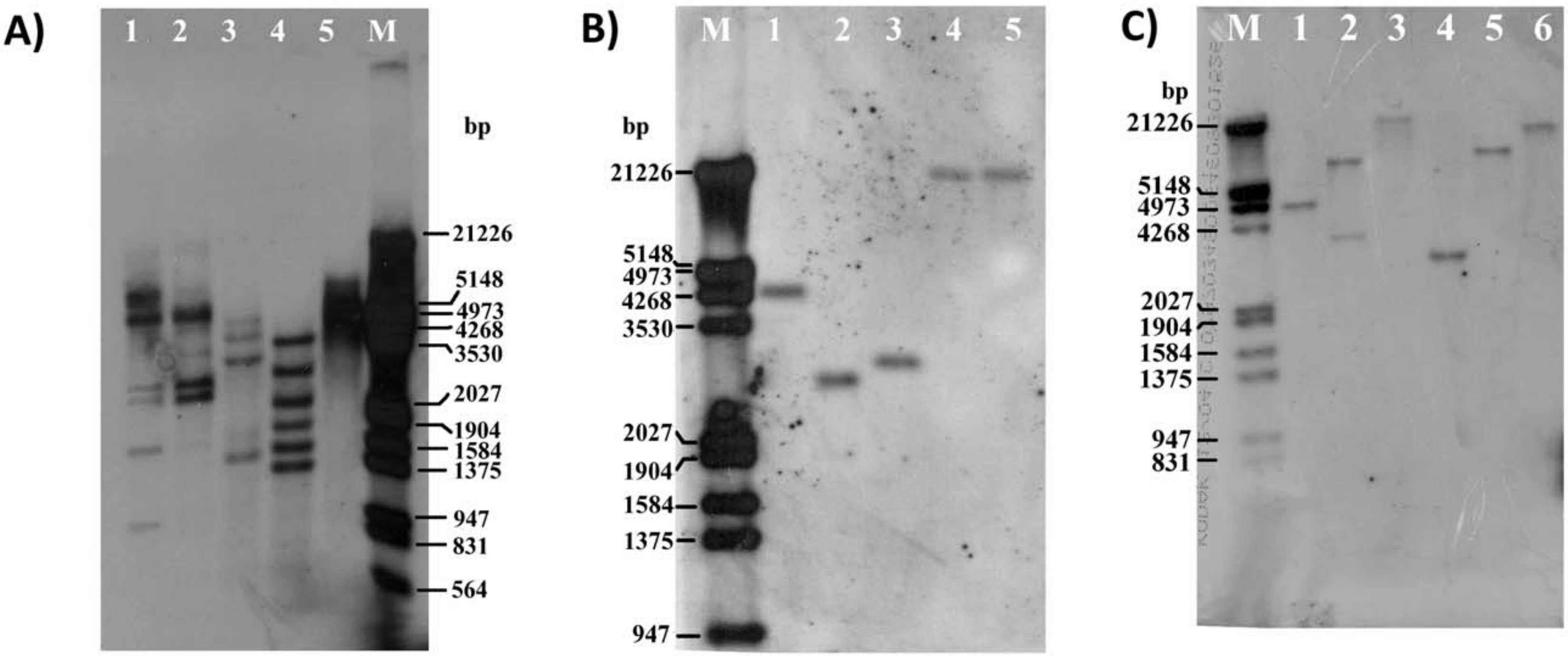

D)

Locus	Probe position (bp)*	Restriction enzyme sites					
		***Eco*RI**	***Eco*RV**	***Bam*HI**	***Hae*III**	***Ban*I**	
ZsMATα copy 1	from 1986 to 2314	2002	352	1894	847, 2309, 2408, 2520	-	
ZsMATα copy 2	from 2029 to 2357	-	399, 1340	1937	341, 2357, 2451, 2563	-	
ZsMATα copy 3	from 1990 to 2318	-	364	1898	306, 2313, 2412, 2524	-	
ZsHML_D copy 1	from 2005 to 2333	2021	317	1913	313, 866, 2836	-	
ZsHML_D copy 2	from 2001 to 2329	-	371, 1312	1909	2832	-	
ZsHML_D copy 3	from 1991 to 2319	2810	365	1899	307	-	
ZsHML copy 1	from 1987 to 2315	2003	353	1895	295, 848, 2310	-	
		***Eco*RI**	***Eco*RV**	***Hind*III**	***Pst*I**	***Pvu*I**	
*ZsMAT***a**	from 1255 to 1839	-	380	2013	-	-	
		***Ava*I**	***Ban*II**	***Sac*II**	***Pst*I**	***Hind*III**	***Bam*HI**
ZsHO copy 1	from 427 to 1059	39	31	-	-	-	-
ZsHO copy 2	from 374 to 1006	-	-	-	229	-	-

*Probe position and restriction site are according to sequences deposited in EMBL database (accession numbers from HG931712 to HG931721).

Figure S3 Southern blot analysis of mating type cassettes and *HO* genes in *Zygoaccharomyces sapae* ABT301^{T}. A) Genomic DNA was digested with *Eco*RI (1), *Eco*RV (2), *Bam*HI (3), *Hae*III (4), and *Ban*I (5) and analyzed with *ZsMTL*α probe in A), with *Eco*RI (1), *Eco*RV (2), *Hind*III (3), *Pst*I (4), and *Pvu*I (5), and analyzed with a *ZsMAT***a** probe in B); with *Ava*I (1), *Ban*II (2), *Sac*II (3), *Pst*I (4), *Hind*III (5), and *Bam*HI (6), and then analyzed with *HO* probe in C). In all plots the DNA molecular weight marker III (Roche) was used as DNA ladder (M). D) The table lists the restriction sites on the *Zygoaccharomyces sapae* mating type cassettes and *HO* genes using the indicated enzymes, and the positions of probes, according to sequences deposited under the accession numbers from HG931712 to HG931721.
