# Supplementary material for: Sex-Determination System in the Diploid Yeast Zygosaccharomyces sapae
Source: G3 (Bethesda). 2014 Jun 1;4(6):1011–25. doi: 10.1534/g3.114.010405 (PMC4065246; doi:10.1534/g3.114.010405)
Supplement: Supporting Information [file supp_4.6.1011_FigureS4.pdf]

```
                                  HO-specific site
CBS 732                           CGCAGCAGTTTAATTTTGTCAAGCCGAAGTGTGGGTTTGTGGAGTGGGTG 50
ZsMATa                            CGCAGCAGTTTAATTTTGTCAAGCCGAAGTGTGGGTTTGTGGAGTGGGTG 50
ZsMATalpha copy 1                 CGCAGCAGTTTAATTTTGTCAAGCCGAAGTGTGGGTTTGTGGAGTGGGTG 50
ZsHML copy 1                      CGCAGCAGTTTAATTTTGTCAAGCCGAAGTGTGGGTTTGTGGAGTGGGTG 50
ZsMATalpha copy 2                 CGCAGCAGTTTAATTTTGTCAAGCCGAAGTGTGGGTTTGTGGAGTGGGTG 50
ZsMATalpha copy 3                 CGCAGCAGTTTAATTTTGTCAAGCCGAAGTGTGGGTTTGTGGAGTGGGTG 50
ZsHML_D copy 1                    CGCAGCAGTTTAATTTTGTCAAGCCGAAGTGTGGGTTTGTGGAGTGGGTG 50
ZsHML_D copy 2                    CGCAGCAGTTTAATTTTGTCAAGCCGAAGTGTGGGTTTGTGGAGTGGGTG 50
ZsHML_D copy 3                    CGCAGCAGTTTAATTTTGTCAAGCCGAAGTGTGGGTTTGTGGAGTGGGTG 50
                                  **************************************************

CBS 732                           GATCAAAGATATGAGCGGGAGAGTTGTATTTAGTTTGTTAAGAGTTGTTG 100
ZsMATa                            GATCAAAGATATGAGCGGGAGAGTTGTATTTAGTTTGTTAAGAGTTGTTG 100
ZsMATalpha copy 1                 GATCAAAGATATGAGCGGGAGAGTTGTATTTAGTTTGTTAAGAGTTGTTG 100
ZsHML copy 1                      GATCAAAGATATGAGCGGGAGAGTTGTATTTAGTTTGTTAAGAGTTGTTG 100
ZsMATalpha copy 2                 GATCAAAGATATGAGCGGGAGAGTTGTATTTAGTTTGTTAAGAGTTGTTG 100
ZsMATalpha copy 3                 GATCAAAGATATGAGCGGGAGAGTTGTATTTAGTTTGTTAAGAGTTGTTG 100
ZsHML_D copy 1                    GATCAAAGATATGAGCGGGAGAGTTGTATTTAGTTTGTTAAGAGTTGTTG 100
ZsHML_D copy 2                    GATCAAAGATATGAGCGGGAGAGTTGTATTTAGTTTGTTAAGAGTTGTTG 100
ZsHML_D copy 3                    GATCAAAGATATGAGCGGGAGAGTTGTATTTAGTTTGTTAAGAGTTGTTG 100
                                  **************************************************

CBS 732                           TAGATTTGTATTTGCATTGATGATGTCTTGTTGGGAGGGGGAAAGTAGTG 150
ZsMATa                            TAGATTTGTATTTGCATTGATGATGTCTTGTTGGGAGGGGGAAAGTAGTG 150
ZsMATalpha copy 1                 TAGATTTGTATTTGCATTGATGATGTCTTGTTGGGAGGGGGAAAGTAGTG 150
ZsHML copy 1                      TAGATTTGTATTTGCATTGATGATGTCTTGTTGGGAGGGGGAAAGTAGTG 150
ZsMATalpha copy 2                 TAGATTTGTATTTGCATTGATGATGTCTTGTTGGGAGGGGGAAAGTAGTG 150
ZsMATalpha copy 3                 TAGATTTGTATTTGCATTGATGATGTCTTGTTGGGAGGGGGAAAGTAGTG 150
ZsHML_D copy 1                    TAGATTTGTATTTGCATTGATGATGTCTTGTTGGGAGGGGGAAAGTAGTG 150
ZsHML_D copy 2                    TAGATTTGTATTTGCATTGATGATGTCTTGTTGGGAGGGGGAAAGTAGTG 150
ZsHML_D copy 3                    TAGATTTGTATTTGCATTGATGATGTCTTGTTGGGAGGGGGAAAGTAGTG 150
                                  **************************************************

CBS 732                           CTTGGAAGTAGAGTTGAGAAGCTTGGGCCAGCAGTGGCGATGGATTGTGT 200
ZsMATa                            CTTGGAAGTAGAGTTGAGAAGCTTGGGCCAGCAGTGGCGATGGATTGTGT 200
ZsMATalpha copy 1                 CTTGGAAGTAGAGTTGAGAAGCTTGGGCCAGCAGTGGCGATGGATTGTGT 200
ZsHML copy 1                      CTTGGAAGTAGAGTTGAGAAGCTTGGGCCAGCAGTGGCGATGGATTGTGT 200
ZsMATalpha copy 2                 CTTGGAAGTAGAGTTGAGAAGCTTGGGCCAGCAGTGGCGATGGATTGTGT 200
ZsMATalpha copy 3                 CTTGGAAGTAGAGTTGAGAAGCTTGGGCCAGCAGTGGCGATGGATTGTGT 200
ZsHML_D copy 1                    CTTGGAAGTAGAGTTGAGAAGCTTGTGCCAGCACTGGCGATGGATTGTGT 200
ZsHML_D copy 2                    CTTGGAAGTAGAGTTGAGAAGCTTGTGCCAGCACTGGCGATGGATTGTGT 200
ZsHML_D copy 3                    CTTGGAAGTAGAGTTGAGAAGCTTGTGCCAGCACTGGCGATGGATTGTGT 200
                                  ************************* ******* ****************

CBS 732                           TGTAGTAGTGTAGTTATCTGATGCGTTAATTGTATTATAGT 241
ZsMATa                            TGTAGTAGTGTAGTTATCTGATGCGTTAATTGTATTATAGT 241
ZsMATalpha copy 1                 TGTAGTAGTGTAGTTATCTGATGCGTTAATTGTATTATAGT 241
ZsHML copy 1                      TGTAGTAGTGTAGTTATCTGATGCGTTAATTGTATTATAGT 241
ZsMATalpha copy 2                 TGTAGTAGTGTAGTTATCTGATGCGTTAATTGTATTATAGT 241
ZsMATalpha copy 3                 TGTAGTAGTGTAGTTATCTGATGCGTTAATTGTATTATAGT 241
ZsHML_D copy 1                    TGTAGTAGTGTACTGATCTGATGGGTTAATTGTATTAGGCT 241
ZsHML_D copy 2                    TGTAGTAGTGTACTGATCTGATGGGTTAATTGTATTAGGCT 241
ZsHML_D copy 3                    TGTAGTAGTGTACTGATCTGATGGGTTAATTGTATTAGGCT 241
                                  ************ * ******** *************  . *
```

Figure S4 Z regions sequence comparisons from *Zygosaccharomyces sapae* strain ABT301[T] and *Zygosaccharomyces rouxii* CBS 732[T]. Aligned Z sequences of eight *Z. sapae* (Zs) mating type cassettes: *ZsMAT*α copies 1, 2, 3, *ZsHML_D* copies 1, 2, and 3, *ZsHML* copy 1, and *ZsMAT***a**.
