# Supplementary material for: Sex-Determination System in the Diploid Yeast Zygosaccharomyces sapae
Source: G3 (Bethesda). 2014 Jun 1;4(6):1011–25. doi: 10.1534/g3.114.010405 (PMC4065246; doi:10.1534/g3.114.010405)
Supplement: Supporting Information [file supp_4.6.1011_FigureS5.pdf]

```
CBS 732                 AGCGATTTGCTGGACGGCGGAGGCGGGGCGGCGGAGGCGGGGCGGGGGCG 50
ZsHML copy 1            AGCGATTTGCTGGACGGCGGAGGCGGGGCGGCGGAGGCGGGGCGGGGGCG 50
ZsMATalpha copy 1       AGCGATTTGCTGGACGGCGGAGGCGGGGCGGCGTAGGCGGGGCGGGGGCG 50
ZsMATa                  AGCGATTTGCTGGACGGCGGAGGCGGGGCGGCGTAGGCGGGGCGGGGGCG 50
ZsMATalpha copy 2       AGCGATTTGCTGGACGGCGGAGGCGGGGCGGCGTAGGCGGGGCGGGGGCG 50
ZsHML_D copy 1          AGCGATTTGCTGGACGGCGGAGGCGGGGCGGCGTAGGCGGGGCGGGGGCG 50
ZsHML_D copy 2          AGCGATTTGCTGGACGGCGGAGGCGGGGCGGCGTAGGCGGGGCGGGGGCG 50
ZsMATalpha copy 3       AGCGATTTGCTGGACGGCGGAGGCGGGGCGGCGTAGGCGGGGCGGGGGCG 50
ZsHML_D copy 3          AGCGATTTGCTGGACGGCGGAGGCGGGGCGGCGTAGGCGGGGCGGGGGCG 50
                        ******************************** *****************

CBS 732                 ATGGTTTTTTCTTGGGGTGGATTCGCTGCTTGGAGACTTTGCCGCCGGGG 100
ZsHML copy 1            ATGGTTTTTTCTTGGGGTGGATTCGCTGCTTGGAGACTTTGCCGCCGGGG 100
ZsMATalpha copy 1       ATGGTTTTTTCTTGGGGTGGATTCGCTGCTTGGAGACTTTGCCGCCGGGG 100
ZsMATa                  ATGGTTTTTTCTTGGGGTGGATTCGCTGCTTGGAGACTTTGCCGCCGGGG 100
ZsMATalpha copy 2       ATGGTTTTTTCTTGGGGTGGATTCGCTGCTTGGAGACTTTGCCGCCGGGG 100
ZsHML_D copy 1          ATGGTTTTTTCTTGGGGTGGATTCGCTGCTTGGAGACTTTGCCGCCGGGG 100
ZsHML_D copy 2          ATGGTTTTTTCTTGGGGTGGATTCGCTGCTTGGAGACTTTGCCGCCGGGG 100
ZsMATalpha copy 3       ATGGTTTTTTCTTGGGGTGGATTCGCTGCTTGGAGACTTTGCCGCCGGGG 100
ZsHML_D copy 3          ATGGTTTTTTCTTGGGGTGGATTCGCTGCTTGGAGACTTTGCCGCCGGGG 100
                        **************************************************

CBS 732                 GCGGGTTTTGTTTTTGCATTTCTCTCAGCGTTGGTTGCAGGTGGGGCTGG 150
ZsHML copy 1            GCGGGTTTTGTTTTTGCATTTCTCTCAGCGTTGGTTGCAGGTGGGGCTGG 150
ZsMATalpha copy 1       GCGGGTTTTGTTTTTGCATTTCTCTCAGCGTTGGTTGCAGGTGGGGCTGG 150
ZsMATa                  GCGGGTTTTGTTTTTGCATTTCTCTCAGCGTTGGTTGCAGGTGGGGCTGG 150
ZsMATalpha copy 2       GCGGGTTTTGTTTTTGCATTTCTCTCAGCGTTGGTTGCAGGTGGGGCTGG 150
ZsHML_D copy 1          GCGGGTTTTGTTTTTGCATTTCTCTCAGCGTTGGTTGCAGGTGGGGCTGG 150
ZsHML_D copy 2          GCGGGTTTTGTTTTTGCATTTCTCTCAGCGTTGGTTGCAGGTGGGGCTGG 150
ZsMATalpha copy 3       GCGGGTTTTGTTTTTGCATTTCTCTCAGCGTTGGTTGCAGGTGGGGCTGG 150
ZsHML_D copy 3          GCGGGTTTTGTTTTTGCATTTCTCTCAGCGTTGGTTGCAGGTGGGGCTGG 150
                        **************************************************

CBS 732                 GGCGGCAGGGTTGGCGGCTTGGGCGTTGGCGGCTTGTGCTTGTGCTTGTG 200
ZsHML copy 1            GGCGGCAGGGTTGGCGGCTTGGGCGTTGGCGGCTTGTGCTTGTGCTTGTG 200
ZsMATalpha copy 1       GGCGGCAGGGTTGGCGGCTTG---------------------------TG 173
ZsMATa                  GGCGGCAGGGTTGGCGGCTTG---------------------------TG 173
ZsMATalpha copy 2       GGCGGCAGGGTTGGCGGCTTG---------------------------TG 173
ZsHML_D copy 1          GGCGGCAGGGTTGGCGGCTTG---------------------------TG 173
ZsHML_D copy 2          GGCGGCAGGGTTGGCGGCTTG---------------------------TG 173
ZsMATalpha copy 3       GGCGGCAGGGTTGGCGGCTTG---------------------------TG 173
ZsHML_D copy 3          GGCGGCAGGGTTGGCGGCTTG---------------------------TG 173
                        *********************                           **

CBS 732                 CTTGTGCTTGCGCTTTGGCGGCGGCGGCGGCGGCAGGACGCCGCAGG 250
ZsHML copy 1            CTTGTGCTTGCGCTTTGGCGGCGGCGGCGGCGGCAGGACGCCGCAGG 250
ZsMATalpha copy 1       CTTGTGCTTGCGCTTTGGCGGCGGCGGCGGCGGCAGGACGCCGCAGG 223
ZsMATa                  CTTGTGCTTGCGCTTTGGCGGCGGCGGCGGCGGCAGGACGCCGCAGG 223
ZsMATalpha copy 2       CTTGTGCTTGCGCTTTGGCGGCGGCGGCGGCGGCAGGACGCCGCAGG 223
ZsHML_D copy 1          CTTGTGCTTGCGCTTTGGCGGCGGCGGCGGCGGCAGGACGCCGCAGG 223
ZsHML_D copy 2          CTTGTGCTTGCGCTTTGGCGGCGGCGGCGGCGGCAGGACGCCGCAGG 223
ZsMATalpha copy 3       CTTGTGCTTGCGCTTTGGCGGCGGCGGCGGCGGCAGGACGCCGCAGG 223
ZsHML_D copy 3          CTTGTGCTTGCGCTTTGGCGGCGGCGGCGGCGGCAGGACGCCGCAGG 223
                        ***********************************************
```

Figure S5 X regions sequence comparisons from *Zygosaccharomyces sapae* strain ABT301[T] and *Zygosaccharomyces rouxii* CBS 732[T]. Partial aligned X sequences of eight *Z. sapae* (Zs) mating type cassettes: *ZsMAT*α copies 1, 2, 3, *ZsHML_D* copies 1, 2, and 3, *ZsHML* copy 1, and *ZsMAT***a**.
